# Novel BPI3Vc-vectored surface displayed fusion and hemagglutinin-neuraminidase antigens elicit broadly neutralizing antibodies in cattle

**DOI:** 10.3389/fimmu.2025.1702440

**Published:** 2026-02-13

**Authors:** Huldah Sang, Tae Kim, Rakshith Kumar, Jayden McCall, Aloysius Abraham, Tsvetoslav Koynarski, Michelle Zajac, Karim W. Abdelsalam, Daniela Hernandez Muguiro, Katherine Bauer, Brandon L. Plattner, Waithaka Mwangi

**Affiliations:** 1 Department of Diagnostic Medicine/Pathobiology, Kansas State University, Manhattan, KS, United States; 2 Research Technology Innovation (RTI) Lab, Brookings, SD, United States; 3 Kansas Veterinary Diagnostic Laboratory, Kansas State University, Manhattan, KS, United States

**Keywords:** antigen surface display, bovine parainfluenza-3 virus, broadly neutralizing antibody, efficacy, mucosal antibody, robust, temperature-sensitive vector, vaccine

## Abstract

Bovine parainfluenza-3 virus (BPI3V) contributes to Bovine Respiratory Disease Complex, causing severe pneumonia and death in cattle, leading to economic losses. Existing BPI3V commercial vaccines, based on genotype A strains, confer protection against some, but not all, genotype A strains and induce low neutralizing antibody titers against genotypes B and C. This study aimed to develop a live vaccine capable of inducing broad protection against diverse BPI3V strains using an attenuated BPI3V vaccine vector based on a genotype C strain. A rescued recombinant BPI3Vc_mutant_GFP virus exhibited a temperature-sensitive attenuated phenotype *in vitro*. Novel Fusion (designated F2) and Hemagglutinin-Neuraminidase (designated HN2) antigens, derived from consensus protein sequences of BPI3V genotypes A, B, and C, were used to develop a recombinant prototype vaccine, designated rBPI3Vc_mut_F2-HN2. The recombinant virus replicated efficiently, displayed the novel antigens on the surface of infected cells, and remained stable over nine *in vitro* passages. Intranasal vaccination of calves with the rBPI3Vc_mut_F2-HN2 virus induced strong systemic and mucosal IgG responses against BPI3V genotypes A, B, and C, which were significantly amplified upon boost, unlike the responses elicited by a commercial vaccine. Notably, sera from calves vaccinated with the rBPI3Vc_mut_F2-HN2 virus had significantly higher (p<0.0001) neutralizing antibodies against BPI3V genotypes A-C compared to the commercial vaccine. The F2-HN2 antigens were critical in eliciting neutralizing antibodies against wild-type BPI3Va and c (p< 0.0001), and BPI3Vb (p<0.001). Upon challenge with wild-type BPI3V genotype C virus, the rBPI3Vc_mut_F2-HN2-vaccinated calves shed the least amount of virus in nasal swabs, had lower viremia, and exhibited minimal pulmonary lesions. Therefore, rBPI3Vc_mut_F2-HN2 virus is a promising vaccine candidate that has potential to confer broad protection against multiple BPI3V strains.

## 1 Introduction

Bovine parainfluenza-3 virus (BPI3V) is a respiratory pathogen that causes disease in cattle and is recognized as one of the etiological agents of Bovine Respiratory Disease Complex (BRDC), a leading cause of morbidity and mortality in feedlot cattle that results in economic losses (1, 2). The virus was first isolated in the United States from nasal discharges of cattle exhibiting respiratory disease symptoms (3). BPI3V is classified within the family Paramyxoviridae and genus *Respirovirus*, with close antigenic and genetic relatedness to human parainfluenza 3 virus and Sendai virus (4). The BPI3V is a single-stranded negative-sense RNA virus with a genome of approximately 16 kb that encodes six structural proteins (Nucleoprotein N, Phosphoprotein P, Matrix M, Fusion F, Hemagglutinin-neuraminidase HN, and Large/polymerase L) and three non-structural proteins (V, C, and D) (1).

The BPI3V is classified into three genotypes: A, B, and C. While genotypes B and C were previously reported in other countries, they are now circulating in U.S. cattle herds (5–7). Current commercial BPI3V vaccines in the U.S. are mainly based on live attenuated genotype A strains, including SF-4 and Kansas/15626/84, which were isolated more than twenty years ago (2, 5, 8–10). These vaccines are effective against some homologous, but not heterologous strains, and their efficacy declines with antigenic drift (5–7). Notably, the number of emerging genomic and antigenic variant field strains, genotypes B and C, was documented to be quite significant in U.S. cattle herds (2, 5, 6). Importantly, the reference sera raised against the genotype A virus contain virus-neutralizing antibodies against some, but not all, genotype A field strains and the neutralizing titers are very low against the genotypes B and C (5). This suggests that there is a decrease in the genetic and antigenic relatedness among the field strains and the currently available vaccines are unable to provide heterologous protection against the divergent strains (11), underscoring the critical need for developing contemporary vaccines capable of conferring broad protection (7).

Vaccines that induce virus-neutralizing antibodies are crucial for protection (1, 12, 13). Although calves receive initial protection through maternal antibodies in colostrum, these antibodies wane by approximately ten weeks of age, leaving calves vulnerable to infections. Therefore, vaccination is essential for long-term immunity (14, 15). Natural BPI3V infections or vaccination can induce neutralizing antibodies (10, 13, 16). Moreover, natural killer cells and cytotoxic T lymphocytes (CTLs) play a role in eliminating BPI3V-infected cells (17). Notably, CTL activity is detected as early as 6–9 days post-infection, coinciding with virus clearance observed in the closely related Sendai virus. This suggests that CTLs play a role in reducing viremia (18, 19). However, it is important to note that, CD8^+^ CTL responses are typically short-lived and less robust compared to virus-neutralizing antibody responses (20).

Intranasal delivery of live BPI3V vaccines is more effective than parenteral methods in eliciting neutralizing antibodies in the respiratory mucosal secretions, thereby reducing viral shedding (21). While some recent BPI3V vaccines are administered subcutaneously (11) or intramuscularly (22), these routes do not mimic natural BPI3V exposure and are, therefore, limited in eliciting mucosal virus-neutralizing antibodies (7). Furthermore, using colostrum-deprived calves to conduct efficacy studies (11) may not take into account the inhibitory effect of maternally derived antibodies on vaccine induced immune response, especially in calves under six months of age. High concentrations of mucosal antibodies are associated with protection from clinical disease following challenge and thus are essential in mitigating initial infection (13, 23). Notably, certain commercially licensed BPI3Va based modified live virus (MLV) vaccines are administered intranasally to circumvent interference from BPI3V-specific maternal antibodies (24–27). While intranasal vaccines stimulate mucosal IgA, IgG is the main antibody present in mucosal fluids of cattle because it is actively transported from the bloodstream to the mucosal surfaces (28, 29).

Rationally designed live vectored vaccines present an attractive platform for developing broadly protective BPI3V vaccines that can be updated to cover emerging strains of interest. These vaccines are characterized by their high immunogenicity, ability to stimulate both cellular and humoral immune responses, suitability for intranasal administration, and are not vulnerable to maternal antibodies. The HN and F surface glycoproteins mediate attachment and entry to host cells, respectively. The extracellular F and HN domains contain BPI3V immunodominant antigens with protective neutralizing epitopes, making them suitable targets for vaccine development (30–32). These targets have been utilized to develop subunit vaccines (22, 32, 33). It has also been reported that calves develop antibody responses against F and HN antigens one week after experimental aerosol infection, and a strong recall response occurs upon re-exposure. Serum antibody titers against these antigens correlate with protection (34). In humans, only the Parainfluenza 3 virus F and HN, but not other antigens, elicit antibodies capable of *in vitro* and *in vivo* neutralization (35). Importantly, F and HN antigen-based subunit vaccines induce protective neutralizing antibodies that confer protection upon challenge (22, 32, 36, 37). We hypothesized that a prototype live-vectored vaccine expressing novel F2 and HN2 antigens, designed using data from all sequenced BPI3V genotypes A-C and delivered intranasally, would confer broad protection.

In this study, a live attenuated Bovine parainfluenza-3 virus genotype c (BPI3Vc) virus vector was developed for intranasal delivery of the novel F2 and HN2 antigens. Safety, tolerability, immunogenicity, and protective efficacy of the recombinant prototype vaccine, designated rBPI3Vc_mut_F2-HN2, was evaluated in Holstein calves challenged with wild-type BPI3Vc virus. Broad neutralization of representative wild-type BPI3V genotypes A, B, and C viruses was evaluated *in vitro*. BPI3V genotype A has previously been used as a vaccine vector, and it retains its infectivity and immunogenicity. It can, therefore, elicit immune responses against self as well as transgene-encoded antigens (38–40). The choice of BPI3V genotype C as a vector was informed by: i) the emergence of BPI3Vc strains in the U.S cattle herds; ii) lack of a protective BPI3Vc vaccine given that the currently available BPI3Va vaccine is poor at conferring cross-protection; and iii) availability of the BPI3Vc genome sequence (5, 7). Insertion of foreign genes into the human parainfluenza virus genome for incorporation and expression is reported to attenuate viral replication (41, 42). The BPI3Vc genome was modified to incorporate foreign glycoproteins. Novel antigens, designated F2 and HN2, derived from the consensus of all F and HN protein sequences from the sequenced BPI3Va-c genomes in the U.S. as of 2019, were rationally designed for multicistronic expression and surface display on infected cells for optimal B-cell recognition. The rBPI3Vc_mut_F2-HN2 virus was evaluated for attenuation, virus stability, multicistronic antigen expression, surface display of the antigens, immunogenicity, and protective efficacy in calves following challenge.

## 2 Materials and methods

### 2.1 Cells

Baby Hamster Kidney cells constitutively expressing T7 RNA polymerase (BSR T7/5 cells) (43), (kindly provided by Dr. Ursula J Buchholz, NIH), were maintained in Glasgow Minimum Essential Medium (Thermo Fisher Scientific) supplemented with 10% fetal bovine serum (FBS), 2% MEM Amino Acid Solution (Thermo Fisher Scientific) and 1% GlutaMax (Thermo Fisher Scientific). Expression of T7 RNA polymerase was maintained by 1 mg/ml Geneticin (Thermo Fisher Scientific) selection on every second passage. Human Embryonic Kidney (HEK) 293A cells (Thermo Fisher Scientific) and Madin–Darby bovine kidney (MDBK) cells (Millipore Sigma, MDBK NBL-1) were maintained in Dulbecco’s Modified Eagle Medium (DMEM) supplemented with 5% FBS, 1% GlutaMAX, 1% Non-essential amino acids (Thermo Fisher Scientific) and 1% Penicillin-streptomycin (Thermo Fisher Scientific).

### 2.2 Wild-type BPI3V viruses

The reverse genetics system for BPI3Va strain Kansas/15626/84 (9) GenBank: AF178654.1) was obtained from the National Institutes of Health (44); (kindly provided by Dr. Shirin Munir and Dr. Ursula J Buchholz). The system was used to generate recombinant BPI3Va, strain Kansas, from cDNA. The wild-type BPI3Vb strain TVMDL15 (GenBank: KJ647284.1) and BPI3Vc strain TVMDL16 (GenBank: KJ647285.1) were obtained from Texas A&M Veterinary Medical Diagnostic Laboratory. Viruses were propagated and tittered in MDBK cells at 37°C with 5% CO_2_.

### 2.3 Monoclonal and polyclonal antibodies

Mouse anti-Flag tag monoclonal antibody (mAb clone M2) specific to the Flag epitope (DYKDDDDK) was commercially sourced (Millipore Sigma, F1804) and used to detect Flag-tagged F2 protein. Mouse anti-His tag mAb clone HIS.H8 targeting the hexa-Histidine epitope (HHHHHH) was also commercially sourced (Invitrogen, MA1-21315) and used to detect His-tagged HN2 protein. The bovine parainfluenza-3 virus antiserum was sourced from USDA National Veterinary Services Laboratories (reagent code 470-BDV), and polyclonal IgGs from the sera were purified in-house by protein-G affinity chromatography (Invitrogen, 10-1242). Rabbit anti-bovine parainfluenza-3 virus genotype-C-specific antiserum was generated by rabbit hyperimmunization (out-sourced services from Robert Sargeant, Infinitech, CA) with wild-type BPI3Vc TVMDL 16 virus heat-inactivated at 56°C for 30 minutes. Polyclonal IgGs from the rabbit sera were purified by protein-G affinity chromatography and validated for binding to BPI3Vc-infected MDBK cells as judged by Immunocytometric analysis. For immunofluorescence and immunocytometric analyses, 4 µg/ml of monoclonal antibodies and 6 µg/ml of purified polyclonal IgG antibodies (pAbs) were used. For Western blots, 1.5 µg/ml of monoclonal antibodies were used.

### 2.4 Design and generation of BPI3Vc plasmid constructs

**Figure 1:**
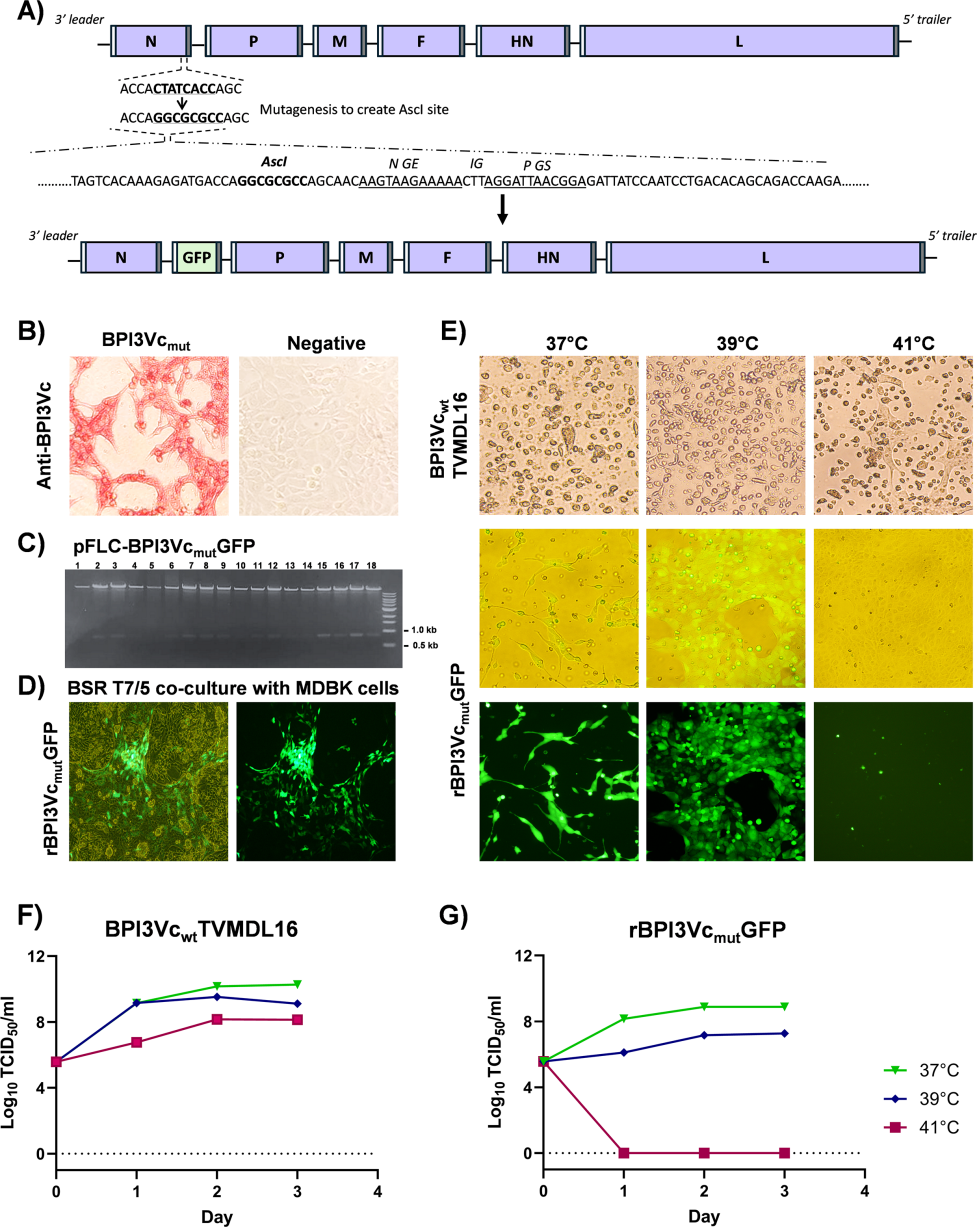
BPI3Vc expression constructs and temperature sensitivity.**(A)** Design of the BPI3Vc construct encoding GFP. Transgenes were cloned, in-frame, between the genes encoding N and P and are flanked by gene start (white bar) and gene end (grey bar) transcription signals, respectively. The genes encoding N and P are separated by an intergenic trinucleotide sequence (CTT), for expression as separate mRNA; **(B)** Assembled BPI3Vc_mut_ virus was authenticated by probing infected MDBK cells using anti-BPI3Vc polyclonal antibody; **(C)** Asci restriction enzyme digestion of the pFLCBPI3Vc_mut_GFP plasmid construct releasing the 15.5kb BPI3Vc vector and 0.8 kb GFP insert; **(D)** Rescued recombinant BPI3Vc_mut_GFP virus in transfected BSR T7/5 cells co-cultured with MDBK cells (left: brightfield view of invaginated BSR T7/5 cells and right: darkfield fluorescent view of infected MDBK cells); and **(E-G)** Recombinant BPI3Vc_mut_GFP virus, but not BPI3Vc_wt_TVMDL 16 virus, is temperature sensitive. **(E)** MDBK cells infected with BPI3Vc_wt_TVMDL16 or rBPI3Vc_mut_GFP virus incubated at 37°C, 39°C or 41°C. GFP expression and extent of infected-monolayer CPE for brightfield view (top) and darkfield fluorescent view (bottom). **(F, G)** Viral TCID_50_ titers up to three days post-infection.

**Figure 2:**
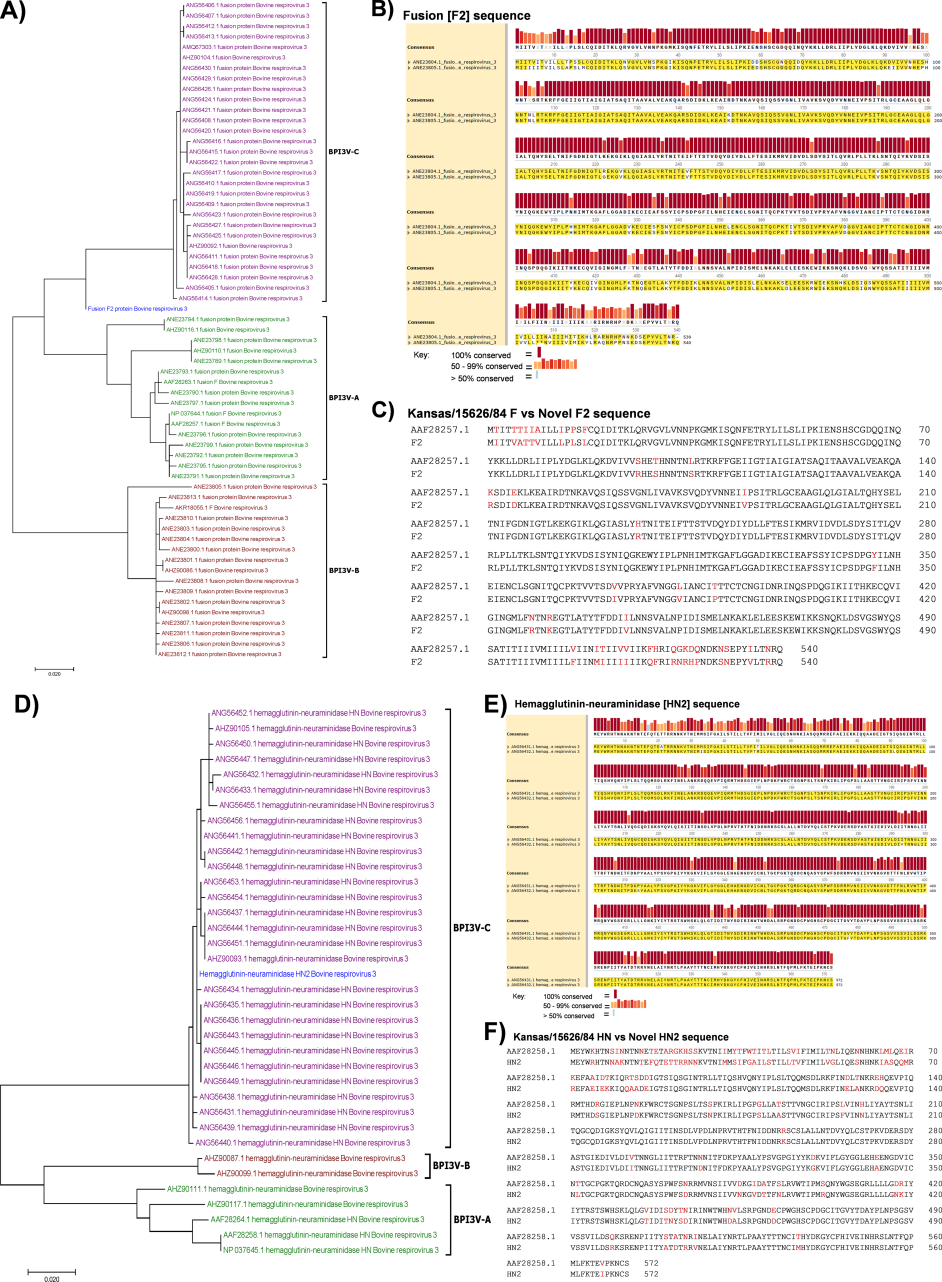
Design of consensus Fusion (F2) and Hemagglutinin-Neuraminidase (HN2) antigens. Clustering of the consensus F2 sequence **(A)** and HN2 sequence **(D)** (shown in blue) in relation to sixty-two BPI3V genotype **(A–C, F)** and thirty-five HN polypeptide sequences, respectively. Conservation levels of F2 **(B)** and HN2 **(E)** consensus sequences. Identity between the BPI3V SF-4 and Kansas/15626/84 vaccine strains F and F2 **(C)**; HN and HN2 **(D)** sequences, respectively.

**Figure 3:**
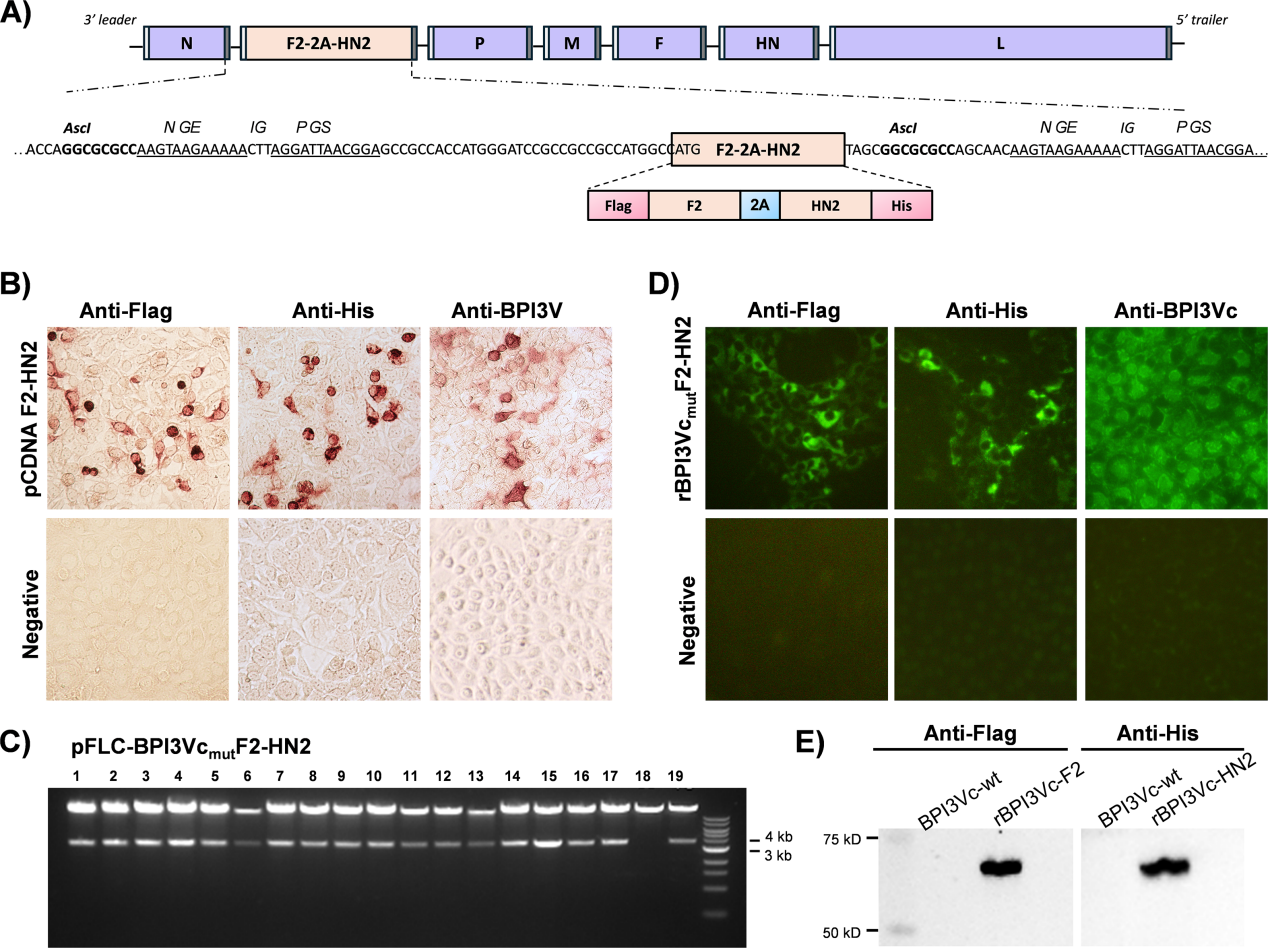
BPI3Vc construct expressing F2-HN2. **(A)** Design of the BPI3Vc construct encoding F2-HN2 genes; **(B)** Expression of pcDNA-encoded F2-HN2 proteins was validated using anti-Flag mAb (to detect F2), or anti-His mAb (to detect HN2), whereas authenticity of the F2-HN2 antigens was confirmed by using anti-BPI3Va pAb; **(C)** Asci restriction enzyme digestion of the pFLCBPI3Vc_mut_F2-HN2 construct; **(D)** Protein expression by MDBK cells infected with the rescued recombinant BPI3Vc_mut_F2-HN2 virus was validated using the same probes as above; and **(E)** Purified virus expression of the individual F2 and HN2 antigens by the multicistronic expression cassette was confirmed by Western Blot analysis using the anti-Flag and the anti-His mAbs. Wild-type BPI3Vc TVMDL 16 virus served as the negative control.

To design the BPI3Vc virus backbone, we selected the BPIV3c strain TVMDL16 (GenBank: KJ647285.1) reference strain in the U.S (7). The antigenomic sequence was used to generate a synthetic virus antigenome that was cloned into the pFLC plasmid vector (obtained from NIH (44) under control of a T7 promoter to generate a construct, designated pFLC-BPI3Vc_mut_ (GenScript), that was used as the backbone for virus rescue. This backbone was modified to create a cloning site for insertion of an additional gene in the non-coding region downstream of the N gene by using primers which introduced an AscI restriction site (**Figure 1**). Novel BPI3V Fusion and Hemagglutinin-Neuraminidase genes, designated F2 and HN2, respectively, were designed and validated for protein expression. Briefly, sixty-two BPI3V genotypes A-C sequences encoding the F protein and thirty-five BPI3V genotypes A-C sequences encoding the HN protein were retrieved from NCBI, representing all the BPI3V genotypes A-C F and HN protein sequences that were available. Multiple sequence alignment was conducted using MEGA 7, which generated consensus sequences for both polypeptides based on a >50% identity threshold. In cases where identity was less than 50%, the most dominant amino acid was selected. If there was neither a consensus nor a dominant amino acid, residues from the BPI3Vc were selected. The final consensus sequences for the F2 and HN2 antigens contained conserved polypeptide sequences from the annotated BPI3V genotypes A-C genomes and were included in generating maximum likelihood phylogenetic trees (**Figure 2**). A multicistronic expression cassette was designed to include the F2 and HN2 protein sequences separated by a 22 amino acid “2A” autocleavable motif (GSGATNFSLLKQAGDVEENPGP) derived from foot-and-mouth disease virus (45). A Flag tag was added, in-frame, at the N-terminus of the F2 polypeptide and a His tag was added, in-frame, at the C-terminus of the HN2 polypeptide (**Figure 3A**). The resulting polypeptide was used to generate a synthetic multicistronic expression gene cassette that was codon-optimized for expression in *Bos taurus* and cloned into the pcDNA 3.1 (+) mammalian expression vector to generate a construct designated pcDNA-F2-HN2 (GenScript). Expression of the F2 and HN2 antigens was validated by transfection of HEK293A cells with the pcDNA-F2-HN2 plasmid construct and immunostaining at 48 hours post-transfection using alkaline phosphatase (AP)-conjugated anti-Flag mAb (Millipore Sigma, F3165), or mouse anti-His mAb (Invitrogen, MA1-21315) followed by an AP-conjugated goat-anti-mouse IgG (Jackson ImmunoResearch, 115-055-003). Authenticity of the expressed F2-HN2 antigens was similarly confirmed by immunostaining of the transfected cells with BPI3V antiserum 470-BDV (National Veterinary Services Laboratories) followed by an AP-conjugated goat anti-bovine IgG (Jackson ImmunoResearch, 101-055-003). Positive signals were detected using ASMX-Fast Red substrate.

The genes encoding GFP, F2-HN2, and an irrelevant *Theileria parva* modified sporozoite surface protein (TMSP) antigen (46) were subcloned into the pFLC-BPI3Vc_mut_ backbone to generate recombinant plasmid constructs, designated pFLC-BPI3Vc_mut_GFP, pFLC-BPI3Vc_mut_F2-HN2, pFLC-BPI3Vc_mut_TMSP, respectively, for virus assembly. Briefly, genes encoding GFP, the F2-HN2 multicistronic expression cassette and TMSP were PCR-amplified using primers shown in **Table 1**. These primers introduced an AscI restriction site at 5’ end, BPIV3c N gene-end, Intergenic region (CTT), BPIV3c P Gene-start, and Kozak sequences upstream of the transgene and an AscI restriction site at the 3’ end of the transgene (**Figure 3A**). The primer design factored in requirements to meet the ‘rule of six’ by the recombinant antigenome sequence, which is critical for successful BPI3V virus assembly and efficient viral replication (47). A previously synthesized pCCI-Brick BPI3Vc_wt_GFP construct (GenScript), pcDNA-F2-HN2 subclone, and a previously validated pcDNA-TMSP67 construct (GenScript) served as PCR templates, respectively. The PCR products were digested with AscI restriction enzyme, gene-cleaned, and subcloned into the AscI restriction site of the pFLC-BPI3Vc_mut_ plasmid backbone (47). Following transformation of MAX Efficiency™ Stbl2™ Competent Cells (Invitrogen, 10268019), colonies were screened by PCR to identify positive clones using vector-specific forward primer and gene-specific reverse primers as shown in **Table 1**. The presence of transgenes in plasmid minipreps of selected positive clones was confirmed by AscI digest followed by Sanger sequencing validation.

**Table 1:** Primers used to amplify genes of interest.

Gene	Primer name	Sequence (5’ → 3’)
GFP	Forward-1	ATGGGATCCACGCGTGCCGCCGCCGCCATGGCCATGGTGAGCAAGGGCGCCGAGCTGTTC
	Forward-2	TAAGAAAAACTTAGGATTAACGGAGCCGCCACCATGGGATCCACGCGTGCCGCCGCCGC
	Forward-3	GATTCTCGTATCGTATCT**GGCGCGCC**AAGTAAGAAAAACTTAGGATTAACGGAGCCGC
	Reverse	CGTGATAGT**GGCGCGCC**GCTAGCTATAGCGGCCGCATTACTATCACTTGTACAGCTCATC
F2-HN2	Forward-1	CGCCAAGTAAGAAAAACTTAGGATTAACGGAGCCGCCACCATGGGATCCGCCGCCGCCATGGCCATGGCCACCATGATCACCATAGTTG
	Forward-2	GATTCTCGTATCGTATCT**GGCGCGCC**AAGTAAGAAAAACTTAGGATTAACGGAGCCGC
	Reverse	CGTGATAGT**GGCGCGCC**GCTAGCTATAGCGGCCGCATCATTACTAGTGGTGATGGTGATG
TMSP	Forward-1	ACTGTGATAATAGTACGCGTGCCGCCGCCGCCATGGCCATGGCCATGACCACCATAGTTG
	Forward-2	TAAGAAAAACTTAGGATTAACGGAGCCGCCACCATGGGATCCACGCGTGCCGCCGCCGC
	Forward-3	GATTCTCGTATCGTATCT**GGCGCGCC**AAGTAAGAAAAACTTAGGATTAACGGAGCCGC
	Reverse-1	AGCTGTAGTGATAATAGTGCGGCCGCATTATCATTACTAGTGGTGATGGTGATGATG
	Reverse-2	CGTACT**GGCGCGCC**GCTAGCTATAGCGGCCGCATTATCATTACTAGTGGTGATGGTGATG
Colony Screening	Vector Forward	AGGGCAGCCTGAATCCAGAGGAGATCAGGATCA
	GFP Reverse	CGTGATAGT**GGCGCGCC**GCTAGCTATAGCGGCCGCATTACTATCACTTGTACAGCTCATC
	F2-HN2 Reverse	CGTGATAGT**GGCGCGCC**GCTAGCTATAGCGGCCGCATCATTACTAGTGGTGATGGTGATG
	TMSP Reverse-1	AGCTGTAGTGATAATAGTGCGGCCGCATTATCATTACTAGTGGTGATGGTGATGATG
Helper N	Forward	ATATACCGCGACAGAATCAAGCTTGCCGCCACCATGTTGAGTCTGTTTGATACATTC
	Reverse	ATGACGATAAGTCCTCGAGCTATTACTTATCGTCATCGTCCTTGTAGTCGCTACTTCCA
Helper P	Forward	ATATACCGCGACAGAATCAAGCTTGCCGCCACCATGGAAGACAATGTTCAAAACAATC
	Reverse	ATGACGATAAGTCCTCGAGTTACTACTTATCGTCATCGTCCTTGTAGTCTTGGGAGCTAA
Helper L	Forward	ATATACCGCGACAGAATCGCGGCCGCGCCGCCACCATGGACACCGAATTCAGCGGTGG
	Reverse	TGACGATAAGTCCTCGAGCTATCATTACTTATCGTCATCGTCCTTGTAGTCATCAATATC

Bold values are AscI restriction site.

### 2.5 Generation and validation of viruses

To assemble and rescue viruses, helper plasmids were first generated and validated for protein expression *in vitro*. Briefly, genes encoding N, P, and L proteins were PCR amplified from the above mentioned pCCI-Brick BPI3Vc_wt_GFP (GenScript) construct encoding the wild-type BPI3Vc virus backbone, using primers that introduced a Flag tag at the 3’ end (**Table 1**). The amplified PCR products were subcloned into pcDNA 3.4 TOPO (Invitrogen) to generate pcDNA-N, -P and -L constructs, respectively. Protein expression was evaluated by immunocytometric analysis using an anti-Flag mAb conjugated to alkaline phosphatase (Millipore Sigma) or the above-mentioned rabbit anti-BPI3Vc pAb. To rescue the virus, 3 µg of the antigenome plasmids pFLC-BPI3Vc_mut_, pFLC-BPI3Vc_mut_GFP, pFLC-BPI3Vc_mut_F2-HN2 or pFLC-BPI3Vc_mut_TMSP, along with 1.6 µg of pcDNA-N, 1.6 µg of pcDNA-P and 0.8 µg of pcDNA-L helper plasmids were co-transfected into the BSR T7/5 cells in 6-well plates using Lipofectamine 3000 reagent (3.75 µl of the Lipofectamine 3000 reagent and 5 µl of P3000 reagent, Invitrogen, L3000015). Cultures were incubated at 37°C for three days, then co-cultured with MDBK cells at a ratio of 1:4 and monitored daily. Rescued viruses were identified by observing cytopathic effects (CPE) 5–10 days later, and then virus stocks were processed from cell pellets and supernatants. Protein expression by the rescued viruses was validated by immunocytometric assay as above or by immunofluorescence assay (IFA). Briefly, MDBK cells were grown in 12-well plates to 80% confluency and infected in triplicates with 10-fold diluted stock rescued BP3Vc_mut_ or the recombinant BPI3Vc_mut_F2-HN2 virus (rBPI3Vc_mut_F2-HN2) for 48–72 h at 37°C. The infected cells were fixed with methanol and probed with the mouse anti-Flag mAb (Millipore Sigma), the mouse anti-His mAb (Invitrogen), or the rabbit anti-BPI3Vc pAb. For IFA, FITC-conjugated secondary antibodies were used at 1:1000 dilution, including goat anti-mouse IgG (Jackson ImmunoResearch) or goat anti-rabbit IgG (Jackson ImmunoResearch). Non-infected cells were used as negative controls. Expression of GFP by the recombinant BPI3Vc_mut_GFP (rBPI3Vc_mut_GFP) virus was evaluated using an Olympus CKX53 fluorescent microscope, and images were captured using an AmScope MU1803-HS digital camera. Protein expression was used to select one best rBPI3Vc_mut_F2-HN2 clone, as judged by IFA, which was expanded in MDBK cells and the virus was purified by sucrose gradient ultra-centrifugation at 20,000 rpm for 2 h over a 30-70% sucrose gradient. The recovered virus was dialyzed twice in 1x PBS, aliquoted, snap-frozen (in 3% FBS, 3.5% sucrose (48), and then stored at -80°C.

Incorporation into the BPI3V virions of the individual Flag-tagged F2 (~60 kDa) and the His-tagged HN2 (~64.5 kDa) antigens in the rBPI3Vc_mut_F2-HN2 virus particles was confirmed by Western blot analysis of sucrose gradient-purified rBPI3Vc_mut_F2-HN2 virus. The wild-type virus BPI3Vc TVMDL16 was used as a negative control. Briefly, approximately 16 µg of the purified rBPI3Vc_mut_F2-HN2 virus was prepared under reducing conditions using NuPage LDS sample buffer (Invitrogen) and NuPage sample reducing buffer (Invitrogen), following the manufacturer’s instructions. A 30 µl aliquot of the reduced and denatured sample was resolved on a 4-12% NuPAGE^®^ Bis-Tris gel (Invitrogen) and then transferred onto a PVDF membrane (Bio-Rad). The membrane was blocked with Starting Block™ Blocking Buffer (Invitrogen) and probed with 1.5 µg/ml HRP-conjugated anti-Flag mAb (Sigma-Aldrich), or 1.5 µg/ml mouse anti-His mAb (Invitrogen), followed by HRP-conjugated goat anti-mouse IgG (Jackson ImmunoResearch) diluted at 1:1000 in blocking buffer. Protein bands were visualized by chemiluminescence using the Clarity Western ECL substrate (Bio-Rad).

### 2.6 Virus growth curves

MDBK cells were grown in six-well plates to 80% confluency and then infected in duplicates at a multiplicity of infection (MOI) of 1 with the following viruses: i) BPI3Vc_wt_ virus (TVMDL16); ii) rBPI3Vc_mut_GFP virus; or iii) rBPI3Vc_mut_F2-HN2 virus. The infected cells were incubated at 37°C, 39°C, or 41°C. Every 24 h, cultures were gently mixed, and a 0.5 ml sample was taken and replaced with an equal volume of fresh media (duplicate samples were pooled for virus titration). For the temperature-sensitivity assay, sampling was done on days 1-3. Virus aliquots were snap-frozen and stored at -80°C. Virus titers were determined for each of the time points by TCID_50_ calculation using the Reed–Muench method (49). For the multicycle replication growth assay, a similar infection setup was carried out and sampling was done on days 1-4.

### 2.7 Flow cytometric analysis

The F2 and HN2 polypeptide sequences were designed to retain their native transmembrane domains, enabling anchoring of the extracellular F2 and HN2 domains on the viral surface, thus mimicking the native presentation of F and HN glycoproteins on the virus, allowing their surface display during replication and packaging of viral progeny in permissive cells. The Flag and His tags that were added, in-frame, to F2 and HN2, respectively, allowed differentiation of the consensus transgene products from their corresponding wild-type F and HN proteins in the BPI3Vc backbone. Surface display of the F2 ectodomain by rBPI3Vc_mut_F2-HN2-infected MDBK cells was evaluated by flow cytometry. Briefly, MDBK cells were grown to 80% confluency, then infected with either the rBPI3Vc_mut_F2-HN2 virus or an irrelevant recombinant BPI3Vc_mut_SARS-CoV-2-Spike (rBPI3Vc_mut_Spike) virus (generated in-house as a COVID-19 prototype subunit vaccine), at an MOI of 1. After incubation at 37°C for 48 h, the infected cell monolayer was washed in 1x PBS, trypsinized using 1x TrypLE select enzyme (Invitrogen), and prepared for surface staining. The cells were aliquoted into 1.0 x10^6^ cells per tube and washed twice with 2% FBS in PBS (blocking buffer). The cells were blocked for 30 minutes at 4°C and then incubated with mouse anti-Flag DyLight 488 mAb (1:250) (Invitrogen, MAI-91878-D488) for 30 min at 4°C in the dark. Afterward, the cells were washed twice and resuspended in the blocking buffer. Data was acquired using BD LSRFortessa^™^ flow cytometer (BD Biosciences, San Jose, CA, USA). The data was analyzed using FlowJo v10. Analysis and gating were guided by data from the negative control irrelevant rBPI3Vc_mut_Spike sample, which did not contain any tag.

### 2.8 Viral RNA extraction and sequencing

MDBK cells were grown in six-well plates to 80% confluency and then infected with the rescued rBPI3Vc_mut_F2-HN2 Passage 1 (P1) virus at an MOI of 1. Infected cells were incubated at 37°C for four days, and the resulting P2 virus was used to infect fresh MDBK cells at a ten-fold dilution. After nine passages, the cell pellets of P2 virus and P9 virus, were collected in 1 ml of Trizol (Thermo Fisher Scientific, 15596026) and processed for total RNA isolation following the manufacturer’s instruction. Briefly, phase separation was achieved using 0.2 ml chloroform (Ambion, AM9730), followed by isopropanol precipitation and ethanol washes. Purified RNA was resuspended in RNAse-free water, reverse transcribed to cDNA using Sequenase 2.0 DNA Polymerase (Life Technologies, 70775Z), Superscript III First Strand Synthesis System (Invitrogen, 18080051) and TaKaRa Taq DNA Polymerase (Clontech, R001a). Sequencing). Sequencing libraries were prepared using the NexteraXT DNA Library Preparation Kit (Illumina, FC-131-1096) and Nextera XT DNA Library Preparation Index Kit (96 Indexes, 384 Samples) (Illumina, FC-131-1002), following the manufacturer’s instructions. Libraries were quantified using a Qubit Flex (ThermoFisher, Q33327). Sequencing was performed on the Illumina MiSeq platform, (Illumina, SY-410-1003), generating paired end reads. Raw sequencing data were processed for quality control and trimming using the Trim Reads function in CLC Genomics Workbench Premium Desktop (Qiagen, 832023), followed by alignment to the reference genome using CLC Genomics Workbench Premium Desktop software. The identity percentage of each gene was obtained by alignment to reference gene and calculation of pairwise comparison. Genomic and read coverage data was obtained from the quality control output of the mapped reads.

### 2.9 Calf immunization

Fifteen male Holstein calves (five months old, sourced from J.R. Livestock Inc, Iowa) were confirmed to be seronegative for BPI3V as determined by sera neutralization tests conducted by Iowa State University Veterinary Diagnostic Laboratory. The calves were then randomly allocated to three groups (n=5) (**Table 2**). Seven days after acclimatization, each calf in group 1 received a priming dose of the rBPI3Vc_mut_F2-HN2 prototype vaccine (5.4 x 10^8^ TCID_50_) followed by a first booster dose of 2.5 x 10^10^ TCID_50_, and a second booster dose of 4.4 x 10^10^ TCID_50_ at weeks 3 and 6 post-priming, respectively. The calves were immunized via intranasal administration of 2 ml of the virus (diluted in Dulbecco’s Modified Eagle Medium, DMEM) using an intranasal mucosal atomization device (Teleflex MAD 300). Calves in group 2 were immunized with a commercial bivalent IBR/PI3 modified live vaccine Bovilis^®^ Nasalgen^®^ IP (Merck Animal Health, Intervet Inc.), administered at the recommended 2 ml dose. Calves in group 3 served as negative controls and received 2 ml of DMEM for the prime and booster doses. The rBPI3Vc_mut_F2-HN2 virus immunogen was back-titrated after administering the priming and booster doses. During the immunization phase, the three groups of calves were housed separately. Their body weights and rectal temperatures were recorded weekly.

**Table 2:** BPI3V calf immunization protocol.

Group	Calf ID	Immunogen (IN)	Prime-boost dose per calf
1. rBPI3Vc_mut_F2-HN2	5661 5662 5664 5697 5701	rBPI3Vc_mut_F2-HN2 virus Live vectored vaccine	5.4 x 10^8^ TCID_50_, 2.5 x 10^10^ TCID_50_, 4.4 x 10^10^ TCID_50_
2. Bovilis Nasalgen IP (Intervet/Merck)	5651 5660 5672 5701 5713	*Commercial MLV cocktail:* Bovine Rhinotracheitis Parainfluenza 3	2 ml, 2 ml, 2ml
3. Sham (DMEM)	5652 5654 5669 5676 5726	DMEM	2 ml, 2 ml, 2ml

(IN), Intranasal route of Immunization.

### 2.10 Evaluation of antibody responses

Coagulated blood and nasal swabs were collected weekly and Indirect ELISA was used to evaluate antibody responses. Briefly, sera were obtained from the coagulated blood by centrifugation, while nasal swabs were collected in 500 µl of 1x PBS. 96 well polystyrene ELISA microplates (Nunc, Thermo Fisher Scientific, 80040LE 0910) were coated in triplicate with 100 µl of heat-inactivated BPI3Va (Kansas strain), BPI3Vb (TVMDL 15 strain), or BPI3Vc (TVMDL16 strain) virus diluted in sodium bicarbonate buffer at final concentration of 1 ug/ml, and the plates were incubated at 4°C overnight. The plates were washed with PBS-Tween 20 (PBST) and then blocked with 300 µl of 1% BSA/PBST for 1 h at 37°C. Following washes, 100 µl of sera diluted 1:100 in the blocking buffer was added in triplicates and incubated for 1 h at 37°C. After washing unbound primary antibodies, 100 µl of HRP-conjugated goat anti-bovine IgG (Jackson ImmunoResearch, 101-035-003) diluted at 1:5000 in blocking buffer was added and the plates were incubated for 1 h at 37°C, washed and developed using 100 µl One Step Ultra TMB (Thermo Fisher Scientific, 34029). The reaction was stopped after ten minutes using 100 µl of 1N hydrochloric acid and read at 450 nm using a BioTek microplate reader (Synergy H1 multi-mode reader). BPI3V-specific IgG responses in each group were presented as mean optical density values. BPI3V-specific IgG responses in nasal swabs were similarly determined, but at 1:4 sample dilution.

### 2.11 Virus neutralization

Neutralization of wild-type BPI3Va (Kansas strain), BPI3Vb (TVMDL 15 strain), and BPI3Vc (TVMDL16 strain) viruses was evaluated using sera from blood collected from the study animals one week after the last boost. Briefly, sera samples were heat-inactivated at 56°C for 30 minutes and 50 µl of each serum sample was serially diluted two-fold in 96-well microtiter plates using DMEM for triplicate test wells. The BPI3Va-c virus stocks were diluted to 300 TCID_50_/ml, and 50 µl of this diluted virus was added to each test well. The serially diluted sera were then incubated with the virus for 2 h at 37°C, and then 10,000 MDBK cells/well were added in a volume of 100 µl. The plates were incubated at 37°C for 72 h and monitored daily for signs of CPE. The presence or absence of virus was confirmed by observing CPE and by IFA using the rabbit anti-BPI3Vc pAb. Virus neutralization titers were expressed as the reciprocal of the highest dilution in which more than 50% CPE or fluorescent staining for the virus was detected (6, 37). Sera obtained from age-matched Holstein calves, undergoing a similar immunization regimen, but with a cognate irrelevant virus construct, rBPI3Vc_mut_TMSP, was used as the control for neutralization assays.

### 2.12 Animal challenge

At sixty days post-priming, the calves were challenged by intranasal instillation of 2.9 x 10^7^ TCID_50_ dose of the wild-type BPI3Vc TVMDL16 virus (challenge virus was back-titrated after administration) (10). The calves were monitored daily for 21 days for clinical signs of respiratory disease, including pyrexia (≥103°F, coughing, mucopurulent nasal discharge, ocular discharge, loss of appetite, and depression as previously described (1). Body weights were recorded on days six and twelve post-challenge, while nasal swabs and uncoagulated blood samples were collected every three days for 18 days. Eight days post-challenge, one calf from the rBPI3Vc_mut_F2-HN2 group, 5661, died due to conditions unrelated to the study (bowel perforation). Three weeks later, the calves were humanely euthanized and transported for necropsy following approved protocols to the Kansas Veterinary Diagnostic Laboratory.

### 2.13 Hematology, nasal and blood viremia

Uncoagulated blood was collected in K2 EDTA-coated tubes on days 0, 3, 6, 9, 12, 15, and 18 post-challenge to evaluate white blood cell counts, platelet counts, and viremia in blood. Blood samples were gently inverted several times after collection, and a Complete Blood Count analysis was performed using an automated hematology analyzer Siemens Advia 2120 at Kansas Veterinary Diagnostic Laboratory. The white blood cell (WBC) and platelet (PLT) counts were recorded and verified by one staff personnel and a veterinary clinical pathologist (DHM) via blood smear examination. To establish a common baseline, the mean WBC or PLT count for each group on DPC 3, 6, 9, 12, 15, and 18 were subtracted from their respective initial values on DPC 0, and the resulting differences were plotted as mean count ratios relative to baseline.

Post-challenge wild-type BPI3Vc virus titers in nasal swabs and blood were determined by sample titration in MDBK cells followed by IFA as previously described (50–52). Briefly, nasal swabs were collected in 500 µl 1x PBS supplemented with 1% penicillin-streptomycin antibiotic, vortexed, centrifuged, filter sterilized and diluted five-fold in DMEM. Blood samples were subjected to three freeze-thaw cycles, centrifuged, and the lysate diluted ten-fold in DMEM. 50 µl of serially diluted nasal swab samples or cell lysate samples were used to infect 96-well MDBK cell monolayers in quadruplicates (for swabs) or nine wells (for lysates). The plates were incubated at 37°C for five days and then fixed with formalin for staining using the rabbit anti-BPI3Vc pAb mentioned above and a FITC-conjugated goat-anti rabbit IgG (Jackson ImmunoResearch). The highest sample dilution where positive MDBK cell staining was observed was used to report the BPI3V titer, as previously reported (53–55). Due to limited amount of nasal swab sample, the endpoint viral dilution titer and not the standard TCID_50_/ml titer determination was conducted.

### 2.14 Necropsy and pathology

To terminate the study, the animals were sedated with xylazine and euthanized by penetrative captive bolt at the study endpoint, and complete necropsy examinations were directed by board-certified anatomic pathologists who were blinded to the animal groupings and treatments. All tissues were examined for evidence of abnormalities, and all gross lesions were recorded, and digital photographs were taken. In addition, digital photographs were collected from all animals of thoracic cavity with thoracic organs *in situ*, and the bilateral lungs after removal from thoracic cavity. The lungs were sampled systematically, and sections of lung that included bronchi were collected and labelled prior to fixation in 10% neutral buffered formalin from the following sites: cranial, middle, and caudal lobes of the right lung and the cranial and caudal lobes of the left lung. The tracheobronchial lymph nodes were measured and recorded. The left cranial, left and right middle lung sections were routinely processed for histopathologic evaluation after 48 h of formalin fixation. Hematoxylin and eosin (H&E) staining of 4-micron sectioned paraffin-embedded tissues was performed by standard laboratory protocols in the histopathology laboratory of the Kansas Veterinary Diagnostic Laboratory. All tissues were evaluated by two veterinary anatomic pathologists (KMB, BLP) and were scored using a modified lung scoring system adapted from previous studies (56, 57). Briefly, all lung sections were given an appropriate histologic morphologic diagnosis for each tissue section, to reflect the observed histologic lesions: lymphoplasmacytic and histiocytic interstitial pneumonia; neutrophilic bronchitis and bronchiolitis; lymphoplasmacytic peribronchiolitis; bronchiolitis obliterans; and bronchial associated lymphoid hyperplasia. The severity from mild, moderate, to severe of each histologic lesion was noted. A summary morphologic diagnosis was then given for each calf based on individual lobe diagnoses, and numerical scores were assigned for each summary morphologic diagnosis by summing the individual lesion scores on a scale reflecting the presence and severity of each lesion within each calf summary morphologic diagnosis (0= normal; 1 = lesion is present; 2 = mild; 3 = moderate; 4 = severe).

### 2.15 Statistical analysis

Data was analyzed using GraphPad Prism 10. For the ELISA data analysis, the significance of differences in mean BPI3V-specific IgG responses between the treatment and control groups during the different sampling time points were analyzed by Two-way ANOVA, followed by Geisser-Greenhouse correction for sphericity and Tukey correction for multiple mean comparisons. For the virus neutralization, clinical outcomes, nasal viral shedding, and histopathology data analysis, the significance of differences between the treatment and control groups during the different time points were analyzed by Two-Way ANOVA followed by Tukey correction for multiple mean comparisons. For the blood viremia analysis, the significance of differences between the treatment and control groups was analyzed by One-Way ANOVA followed by Dunnett’s T3 correction for multiple comparisons. Statistical differences with significance levels of p < 0.05 was considered significant.

## 3 Results

### 3.1 Recombinant BPI3Vc is temperature sensitive and expresses transgene

A modified BPI3V genotype-C (BPI3Vc) virus backbone based on the TVMDL16 isolate was designed with an AscI restriction site in the non-coding region of the N gene for cloning of transgenes, and a synthetic gene was used to generate a plasmid construct designated pFLC-BPI3Vc_mut_ (**Figure 1A**). A mutant virus, designated BPI3Vc_mut_, was rescued and validated using rabbit anti-BPI3Vc pAb (rabbit was hyperimmunized with heat inactivated BPI3Vc) (**Figure 1B**). The ~0.8 kb gene encoding GFP was subcloned into pFLC-BPI3Vc_mut_ plasmid (**Figure 1C**) and a recombinant virus, rBPI3Vc_mut_GFP, that was recovered using the resultant plasmid pFLC-BPI3Vc_mut_GFP, expressed GFP (**Figure 1D**). The virus displayed a temperature sensitive attenuated phenotype *in vitro* (**Figures 1E-G**). Notably, replication of the rBPI3Vc_mut_GFP virus was similar to the wild-type BPI3Vc TVMDL16 virus at 37°C, resulting in CPE with peak titers of 8.9 TCID_50_/ml and 10.3 TCID_50_/ml, respectively, (**Figures 1E-G**). However, the replication efficiency of the rBPI3Vc_mut_GFP virus declined at 39°C and reduced at 41°C (**Figure 1G**). In contrast, the onset of replication, CPE, and peak replication titers observed in the cells infected with the BPI3Vc wild-type virus remained relatively unchanged even at 41°C, with titers reaching 8.14 TCID_50_/ml (**Figures 1E, F**).

Novel BPI3V Fusion and Hemagglutinin-Neuraminidase polypeptides, designated F2 and HN2, respectively, based on consensus F protein sequence from sixty-two BPI3V genotypes A-C genomes and consensus HN protein sequence from thirty-five BPI3V genotypes A-C genomes, were generated (**Figures 2A, D**). The F2 polypeptide sequence was 74.1% conserved with all the 62 F protein sequences from the BPI3Va, b and c strains, while the HN2 polypeptide sequence was 77.5% conserved with all the 35 HN protein sequences from the BPI3Va, b and c strains (**Figures 2B, E**). Importantly, the F2 sequence displayed 92.8% and 93.5% identity with the F sequence of BPI3Va vaccine strains Kansas/15626/84 (**Figure 2C**) and SF-4 (data not shown), respectively. Conversely, the HN2 sequence displayed 85.8% and 86.5% identity with the HN sequence of BPI3Va vaccine strains Kansas/15626/84 (**Figure 2F**) and SF-4 (data not shown), respectively.

**Figure 4:**
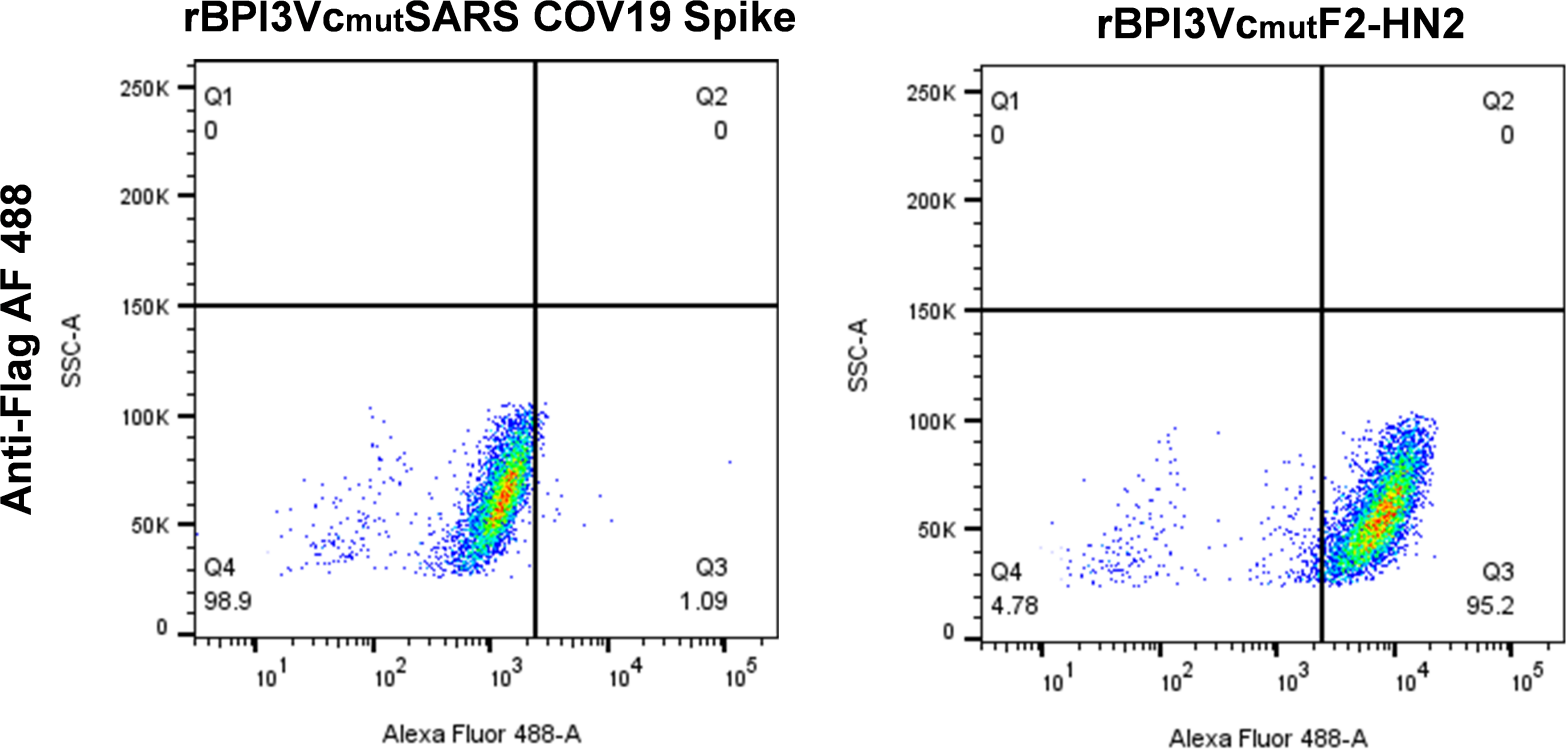
Surface display of F2. Display of F2 on the surface of MDBK cells infected with the rescued rBPI3Vc_mut_F2-HN2 virus was validated using anti-Flag AF 488 mAb (to detect F2) by Flow cytometric analysis. A cognate irrelevant virus, rBPI3Vc_mut_Spike virus, served as the negative control.

A codon-optimized synthetic gene encoding the F2 and the HN2 polypeptides, separated by the 2A auto-cleavable motif, was used to generate a plasmid construct, designated pcDNA-F2-HN2. The construct expressed the encoded antigens that were recognized by anti-tag mAbs and more importantly, by BPI3V-specific serum (**Figure 3B**). The ~3.6 kb gene encoding the F2-HN2 multicistronic expression cassette was subcloned into pFLC-BPI3Vc_mut_ plasmid (**Figure 3C**) and used to generate a recombinant virus, rBPI3Vc_mut_F2-HN2, which incorporated and expressed the encoded proteins as judged by IFA and Western blot assay of purified virus, using Flag- and His-tag-specific mAbs and authenticity of the antigens was validated using rabbit anti-BPI3Vc pAb (**Figures 3D, E**). Rescued rBPI3Vc_mut_F2-HN2 virus, unlike an irrelevant negative control rBPI3Vc_mut_Spike, displayed the F2 antigen on the surface of MDBK infected cells (**Figure 4**).

### 3.2 Recombinant BPI3Vc is stable

**Figure 5:**
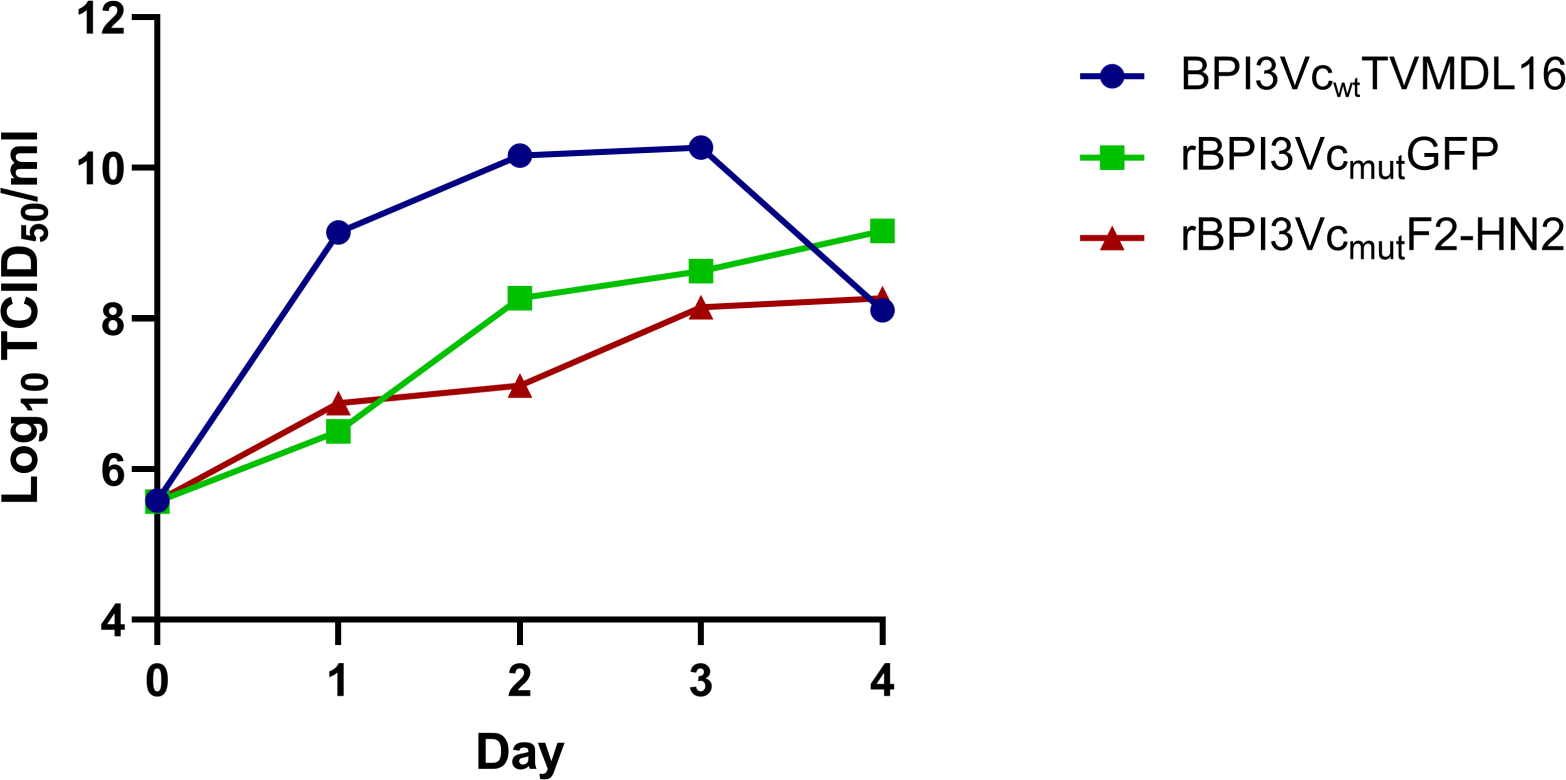
Characterization of the rBPI3Vc_mut_F2-HN2 virus. Multicycle replication growth curve of the BPI3Vc_wt_TVMDL16 wild-type virus, rescued rBPI3Vc_mut_GFP, and rBPI3Vc_mut_F2-HN2 viruses in MDBK cells infected at an MOI of 1 and incubated at 37°C for four days. Virus aliquots were sampled every 24 h and quantified by TCID_50_ virus titration.

Analysis of virus replication curves showed that the presence of transgenes did not inhibit virus replication (**Figure 5**). Multicycle replication of the rBPI3Vc_mut_F2-HN2 virus in MDBK cells at 37°C was similar to that of the rBPI3Vc_mut_GFP virus. By day four, the virus titer for the rBPI3Vc_mut_F2-HN2 was 8.27 log_10_ TCID_50_/ml, while rBPI3Vc_mut_GFP had a 9.16 log_10_ TCID_50_/ml titer. Both titers were comparable to the 8.11 log_10_ TCID_50_/ml titer recorded for the wild-type BPI3Vc TVMDL16 virus (**Figure 5**), suggesting that the presence of the ~0.8 kb GFP insert or the ~3.6 kb F2-HN2 transgenes attenuates viral replication but does not affect overall virus titer.

To evaluate the stability of F2-HN2 antigen expression in MDBK cells, the rBPI3Vc_mut_F2-HN2 virus was serially passaged multiple times *in vitro* and the full-length viral antigenome of the progenies were sequenced. After nine passages, reads covering the N, P, M, F genes of the BPI3Vc backbone and the gene encoding the F2-HN2 polypeptide remained identical to the original sequence and the data from P2 progenies. However, minor point mutations were identified in the HN gene (K254R) and L gene (S2220Y, S2221L) of passage nine virus (**Tables 3A**, **B**). Therefore, passage of the rBPI3Vc_mut_F2-HN2 virus did not significantly alter the antigen sequence or the overall virus antigenome sequence, underscoring the stability of the vector *in vitro* and its potential suitability as a vaccine replicon.

**Table 3A:** rBPI3Vc_mut_F2-HN2 viral RNA sequence at Passage 2.

Gene	Identity percentage	Read coverage	Genomic coverage
*N*	100	18704.25	99.94
*F2-HN2*	100	11271.62	100
*P*	100	4944.43	99.95
*M*	100	2833.28	99.81
*FHN*	100	2917.46	100
*L*	99.99	942.47	99.95

**Table 3B:** rBPI3Vc_mut_F2-HN2 viral RNA sequence at Passage 9.

Gene	Identity percentage	Read coverage	Genomic coverage
*N*	100	1553.32	99.85
*F2-HN2*	100	9417.82	100
*P*	100	898.52	99.81
*M*	100	66520.04	100
*FHN*	99.97	302.11	99.95
*L*	99.94	361.48	99.76

### 3.3 rBPI3Vc_mut_F2-HN2 virus elicited systemic and mucosal antibody responses

**Figure 6:**
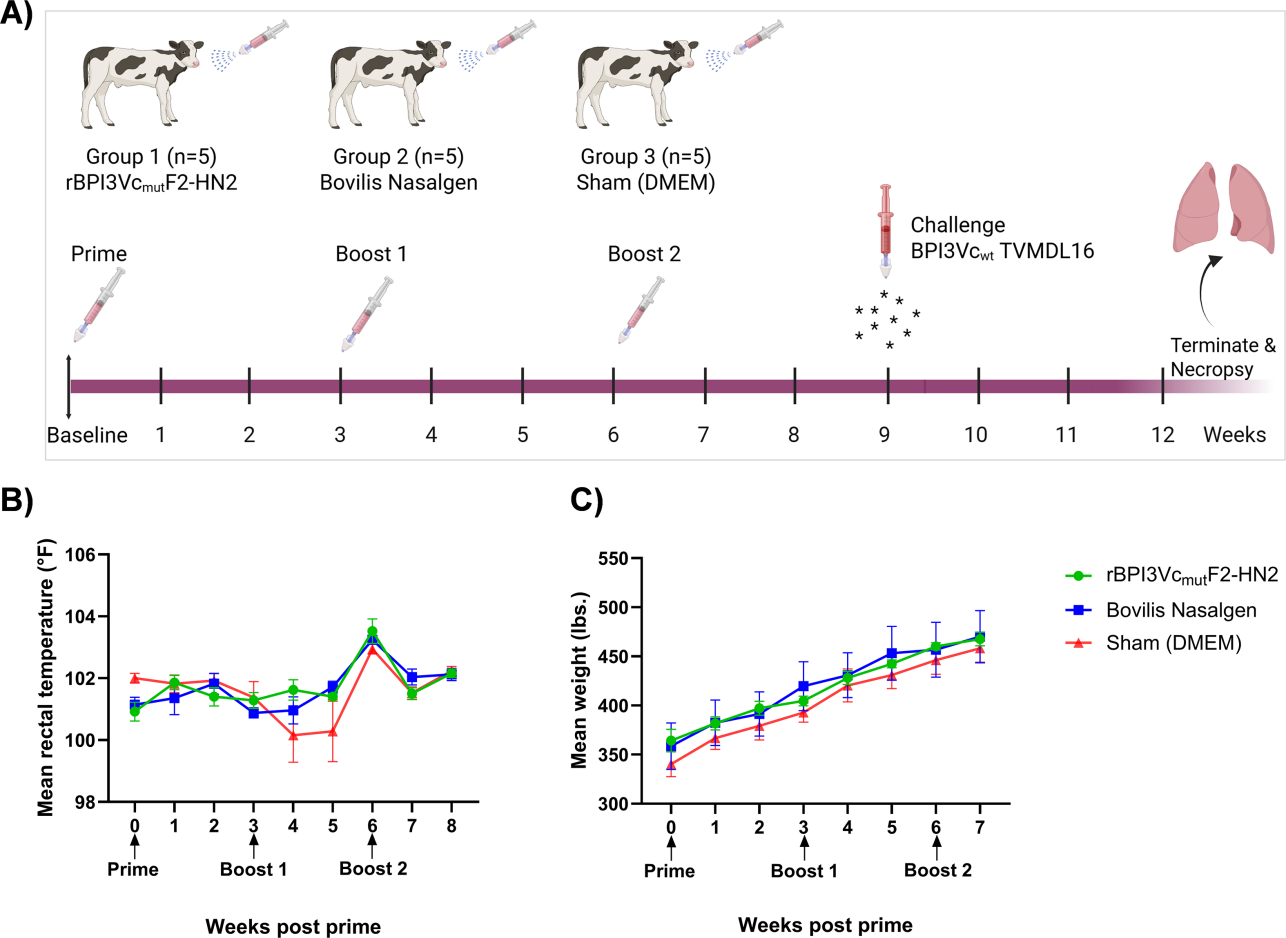
Animal study. **(A)** Immunization design and timeline. Calves were assigned to three treatment groups: 1) rBPI3Vc_mut_F2-HN2 vaccine; 2) Commercial Bovilis Nasalgen vaccine; and 3) Sham-treated with DMEM media. Following one week acclimation, the calves received intranasal instillation of the priming dose followed by booster doses three weeks apart. All calves were challenged nine weeks post-priming by intranasal administration of the wild-type BPI3Vc TVMDL16 virus and the study was terminated 13 wks after inititation of the study. Sampling for whole blood (for PBMCs and sera), and nasal swabs was done weekly; **(B)** Mean rectal temperatures for each experimental group; and **(C)** mean weight gain for each group were measured weekly. Data is presented as group mean ± SEM.

**Figure 7:**
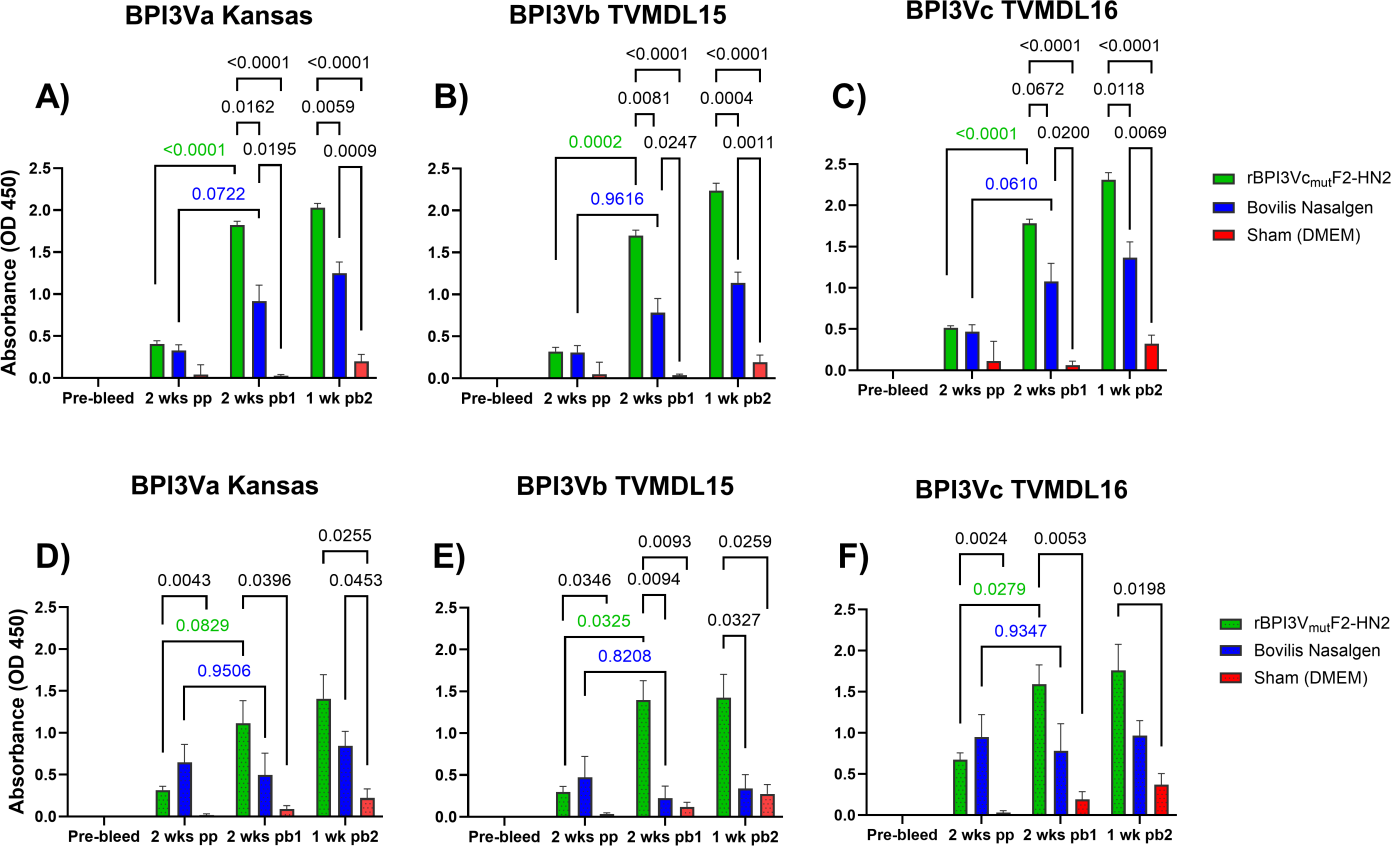
Antibody response. Recognition of inactivated wild-type BPI3Va, b, and c virus by IgGs in sera **(A-C)** or nasal swabs **(D-F)** was evaluated by Indirect ELISA using samples collected before immunization (Pre-bleed), at two weeks post-priming (2 wks pp), two weeks post-boost 1 (2 wks pb1) and one week post-boost 2 (1 wk pb2) from all the treatment groups. Bars represent group means + SEM and the statistical differences in group means are shown.

The rBPI3Vc_mut_F2-HN2 virus was well tolerated by five-month-old Holstein calves following homologous prime-boost intranasal immunization, and no adverse clinical side effects were observed. The calves remained healthy, maintained relatively consistent body temperatures, and showed steady body weight gain during the immunization phase (**Figures 6A-C**). Similar outcomes were observed in the positive control calves which received the commercial Bovilis Nasalgen PI3 vaccine as well as the sham-treated negative controls (**Figures 6A-C**). More importantly, the rBPI3Vc_mut_F2-HN2 virus elicited strong BPI3V-specific systemic and mucosal IgG responses that were amplified after boosting (**Figure 7**). Notably, two weeks post-priming, all the calves immunized with the rBPI3Vc_mut_F2-HN2 virus or the Bovilis Nasalgen PI3 vaccine, but not the sham-treated calves, seroconverted (**Figure 7**). The vaccinees had similar serum and mucosal IgG responses against heat-inactivated BPI3Va (Kansas strain), BPI3Vb (TVMDL15 strain) and BPI3Vc (TVMDL16 strain) wild-type viruses (**Figures 7A-F**). Compared to sham controls, calves immunized with the rBPI3Vc_mut_F2-HN2 virus elicited significantly higher (p<0.0001) BPI3Va-c-specific serum IgG responses two weeks after both the first boost (mean OD values of 1.822, 1.701, and 1.781, respectively) and the second boost (mean OD values of 2.027, 2.236, and 2.310, respectively) (**Figures 7A-C**). Importantly, these responses were significantly higher than those detected in the Bovilis Nasalgen PI3 vaccinees at the corresponding timepoints, where lower BPI3Va-c-specific titers were detected even after the second boost (mean OD values of 0.9183, 0.7819, and 1.075, respectively) (**Figures 7A-C**). Notably, the rBPI3Vc_mut_F2-HN2 virus primed BPI3Va-c-specific serum IgG responses that were significantly amplified following the first, but not the second boost. Specifically, a single boost increased the mean OD values from 0.4065 to 1.822 (BPI3Va), 0.3193 to 1.701(BPI3Vb), and 0.5141 to 1.781 (BPI3Vc), corresponding to statistically significant differences of p<0.0001, p=0.0002, and p<0.001, respectively (**Figures 7A-C**). Similar outcomes were observed in the Bovilis Nasalgen PI3 vaccinees, but boosting had a much lower effect (**Figures 7A-C**).

Interestingly, intranasal immunization of calves with the rBPI3Vc_mut_F2-HN2 virus primed BPI3Va-c-specific mucosal IgG responses that were significantly recalled after boosting (**Figures 7D-F**). Specifically, compared to the sham treatment, the rBPI3Vc_mut_F2-HN2 virus primed significant mucosal IgG responses against BPI3Va (p=0.0043), BPI3Vb (p=0.0346), and BPI3Vc (p=0.0024) (**Figures 7D-F**). Similar to the outcome observed for serum IgG responses, the first, but not the second dose had significant booster effect on BPI3Va-c-specific mucosal IgG responses. Mean OD values increased from 0.3154 to 1.115 (BPI3Va), 0.2967 to 1.397 (BPI3Vb), and 0.6740 to 1.590 (BPI3Vc), corresponding to statistically significant differences of (p=0.0829, p=0.0325, and p=0.0279, respectively) (**Figures 7D-F**). Even though the Bovilis Nasalgen PI3 vaccine primed slightly higher BPI3Va-c-specific mucosal IgG responses compared to the responses primed by the rBPI3Vc_mut_F2-HN2 virus, boosting with the commercial vaccine did not have a booster effect (**Figures 7D-F**). Overall, the rBPI3Vc_mut_F2-HN2 virus had significantly higher booster effect on both serum as well as mucosal IgG responses compared to the Bovilis Nasalgen PI3 vaccine (**Figures 7A-F**). Surprisingly, the Bovilis Nasalgen PI3 vaccine had a subtle booster effect on serum, but not on mucosal IgG responses (**Figures 7A-F**).

### 3.4 rBPI3Vc_mut_F2-HN2 prototype vaccine elicited broadly neutralizing antibodies

**Figure 8:**
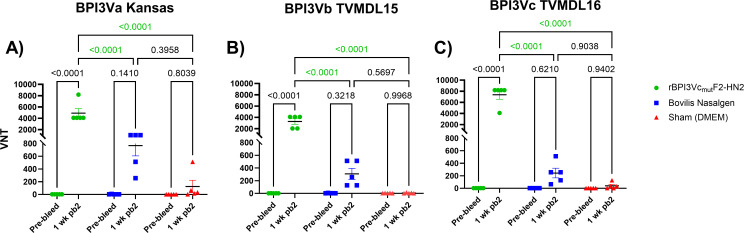
Virus neutralization. Titers of virus neutralization antibodies in the sera from rBPI3Vc_mut_F2-HN2 vaccinees, Bovilis Nasalgen positive controls, and the sham treatment group were determined by neutralization assay against representative BPI3Va, b and c strains **(A–C)** using sera from blood samples collected at one-week post-boost-2. Sera from cognate pre-bleed served as the baseline. Bars represent mean group titers ± SEM.

Immunization of calves with the rBPI3Vc_mut_F2-HN2 prototype vaccine induced antibody responses that neutralized wild-type BPI3Va (Kansas strain), BPI3Vb (TVMDL 15 strain), and BPI3Vc (TVMDL16 strain) viruses as judged by virus neutralizing assay (VNA) using sera from blood collected one week after the second boost (**Figure 8**). Specifically, the rBPI3Vc_mut_F2-HN2 virus induced significantly higher (p<0.0001) BPI3Va-c neutralizing antibody titers (1:4096 -1:8192; 1:2048 - 1:4096; and 1:4096 - 1:8192, respectively), compared to the response detected in sera from the Bovilis Nasalgen PI3 vaccinees (1: 256 - 1:1024; 1:128 - 1:512; and 1:64 - 1:512, respectively) as well as the sham controls (**Figures 8A-C**).

### 3.5 F2-HN2 antigens were critical for eliciting neutralizing antibody

**Figure 9:**
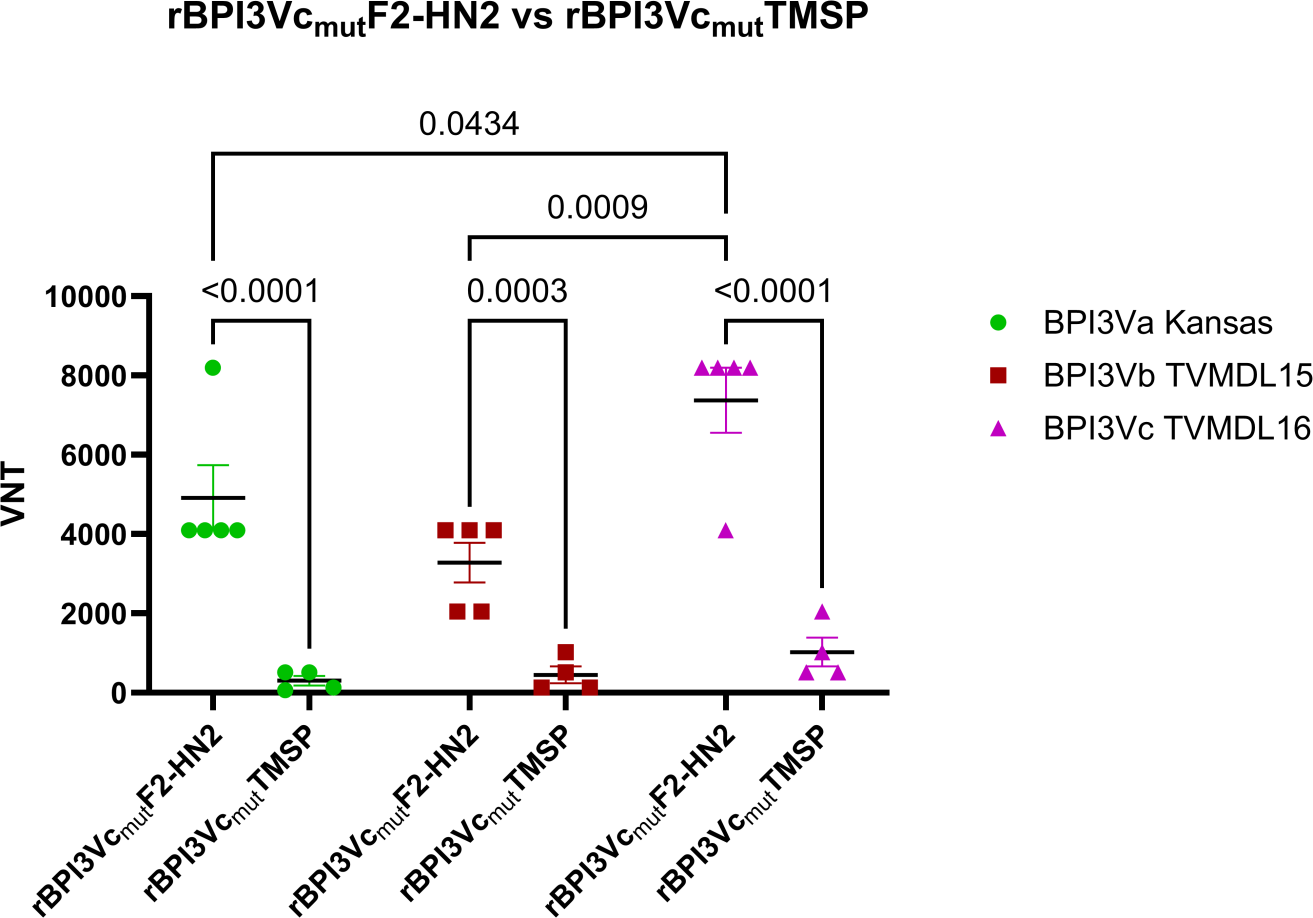
The F2-HN2 antigens were responsible for the robust broadly neutralizing antibodies. Virus neutralization assays were used to determine BPI3Va-, b- and c-specific neutralization titers in sera from blood collected from the rBPI3Vc_mut_F2-HN2 vaccinees at ten days post-boost-2 and compared to titers in sera from cattle immunized with the control rBPI3Vc_mut_TMSP virus construct. Mean group titers are represented by bars ± SEM. .

The BPI3Va-c neutralizing antibodies primed and expanded by the rBPI3Vc_mut_F2-HN2 prototype vaccine were mainly elicited by the F2-HN2 antigens. Calves vaccinated with the rBPI3Vc_mut_F2-HN2 vaccine elicited significantly higher virus neutralization titers against wild-type BPI3Va (1:64 - 1:512), BPI3Vc (1:512 -1:2048) (p< 0.0001), as well as BPI3Vb (1:128 - 1:1024) (p<0.001), compared to titers elicited by a cognate control virus construct, rBPI3Vc_mut_TMSP, expressing an irrelevant *Theileria parva* sporozoite protein (TMSP) (**Figure 9**). Notably, the neutralizing antibody titers observed in the sera from the rBPI3Vc_mut_TMSP vaccinees against wild-type BPI3Va-c, resembled the titers observed in the sera from the Bovilis Nasalgen PI3 vaccinees (**Figure 8**).

### 3.6 Post-challenge clinical signs

**Figure 10:**
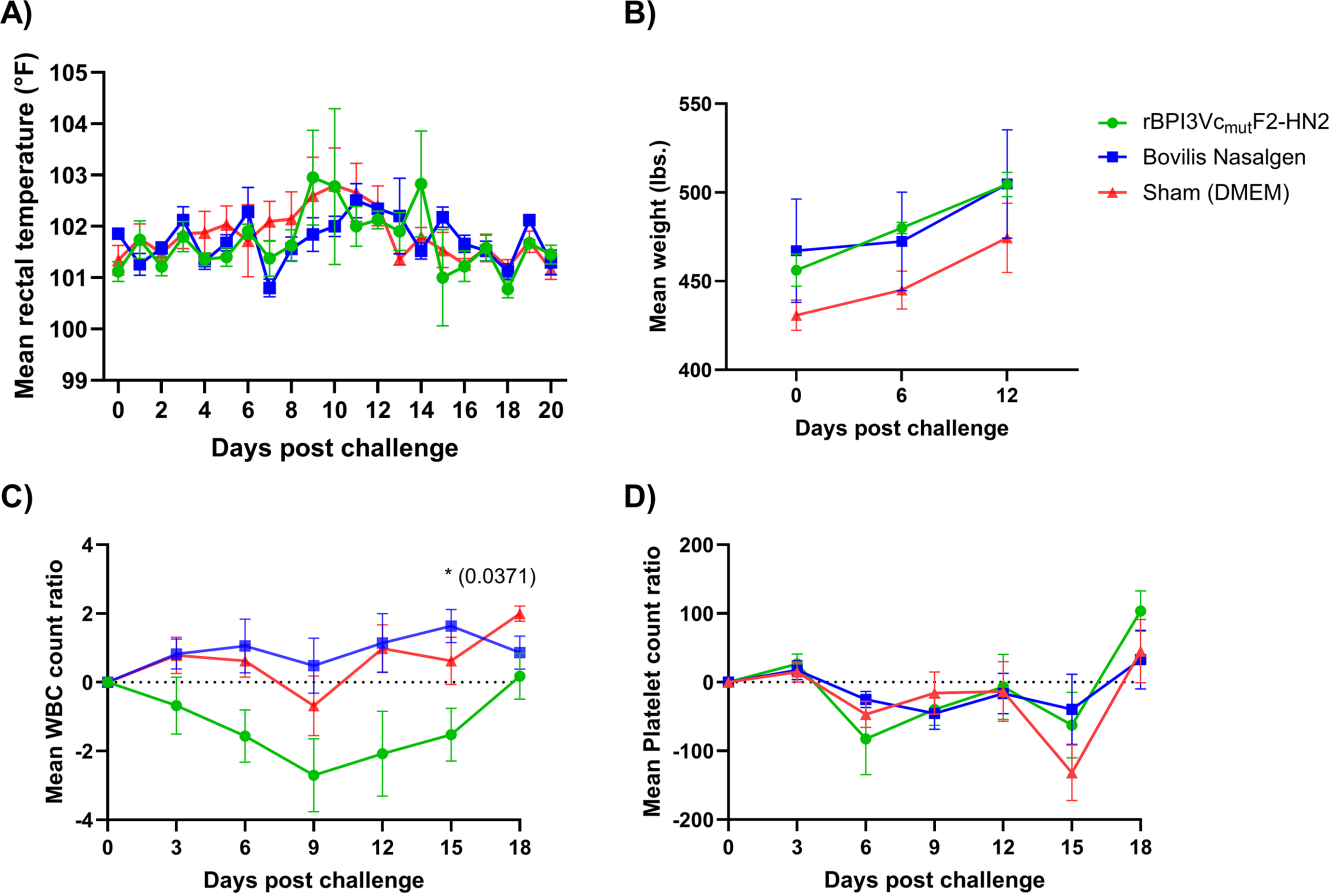
Clinical outcomes post-challenge. **(A)** Mean rectal temperature change for each group recorded every day post-challenge is plotted; **(B)** mean gain in body weight for each group recorded on the day of challenge, six- and twelve-days post-challenge is plotted; **(C)** Mean change in white blood cell; and **(D)** change in platelet count change ratios for each group quantified on every third day post-challenge is plotted. Data is presented as group mean ± SEM.

Following intranasal challenge of all the calves with wild-type BPI3Vc TVMDL 16 virus, some calves developed one or more observable signs of clinical disease, including coughing, nasal discharge, ocular discharge, loss of appetite, depression, and pyrexia. However, no significant differences were observed between the treatment groups. An increase in basal body temperature was observed in all the treatment groups, with peak mean temperatures recorded on day 9 at 103°F, day 10 at 102.5°F, and day 11 at 102.8°F for the rBPI3Vc_mut_F2-HN2 vaccinees, the Bovilis Nasalgen PI3 vaccinees, and the sham treatment controls, respectively. There was no significant difference in temperature fluctuations between the vaccinated and the non-vaccinated negative controls (**Figure 10A**). Post challenge, the mean weight of the rBPI3Vc_mut_F2-HN2 vaccine group increased from 456.2 lbs. to 504.5 lbs., a gain of 48.3 lbs.; while the mean weight of the Bovilis Nasalgen PI3 vaccinees increased from 467.2 lbs. to 521.5 lbs., a gain of 54.3 lbs. In contrast, the sham treatment controls had the lowest mean weight gain, from 430.8 lbs. to 474.4 lbs., a gain of 43.6 lbs. (**Figure 10B**).

All treatment groups experienced a decrease in WBC counts, with onset detected on day 3 post-challenge in the rBPI3Vc_mut_F2-HN2 vaccinated calves and on day 6 post-challenge in the Bovilis Nasalgen vaccinated calves and the sham-treated calves (**Figure 10C**). The greatest drop in WBC counts happened on day 9 post-challenge, from 9,040/µl on DPC 0 to 6,700/µl in the rBPI3Vc_mut_F2-HN2 vaccinees; 8,720/µl on DPC 3 to 8,380/µl in the Bovilis Nasalgen vaccinees; and 7,540/µl on DPC 3 to 6,860/µl in the sham-treated negative control calves. Surprisingly, the rBPI3Vc_mut_F2-HN2 vaccinees had the lowest mean WBC counts throughout the challenge phase compared to the Bovilis Nasalgen PI3 vaccinees and the sham negative controls (**Figure 10C**). The Bovilis Nasalgen PI3 vaccinees had significantly higher mean WBC counts (p=0.0371) compared to the rBPI3Vc_mut_F2-HN2 vaccinees on day 15 post-challenge (**Figure 10C**). This reduction, however, was within the normal range of 4,700-11,400/µl in healthy calves of up to six months of age (58). The Bovilis Nasalgen PI3 vaccinees and the negative controls recovered by day 12 post-challenge, while recovery in the rBPI3Vc_mut_F2-HN2 vaccinees occurred by day 18 (**Figure 10C**).

Platelet counts decreased across all treatment groups by day 6 post-challenge (**Figure 10D**). However, by day 12, the rBPI3Vc_mut_F2-HN2 vaccinees exhibited a higher recovery rate (from 290 K/mm^3^ to 372.7 K/mm^3^) compared to the negative controls (from 322.4 K/mm^3^ to 414.4 K/mm^3^). By day 15 post-challenge, the mean platelet count in the negative control group, compared to the vaccine treatment groups, declined to the lowest level recorded throughout the challenge phase, reaching 237.6 K/mm^3^. This reduction, however, was within the normal range of 200–590 K/mm^3^ in healthy calves from five months of age (58). By day 18 post-challenge, all the vaccinees and the negative controls had recovered, with the rBPI3Vc_mut_F2-HN2 vaccine treatment group recording the highest mean platelet counts (483.25 K/mm^3^) (**Figure 10D**). (Notably, sporadic platelet clumps were observed, which may affect the actual recorded counts).

### 3.7 rBPI3Vc_mut_F2-HN2 vaccinees had lower viral loads

**Figure 11:**
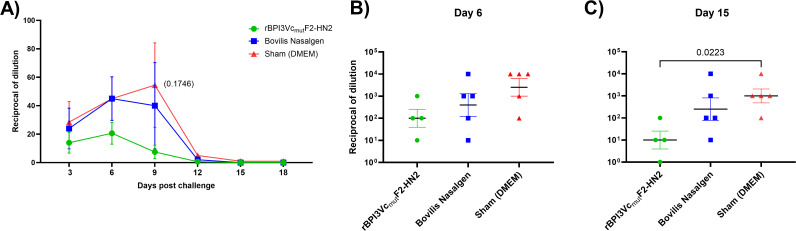
Post-challenge Viremia. **(A)** Nasal shedding was determined in nasal swabs collected from treatment and control calves from 3–18 days post-challenge. Mean group dilutions are plotted ± SEM. Viremia was determined in blood samples collected from treatment and control calves on day 6 **(B)** and day 15 **(C)** post-challenge. Mean group dilutions are represented by the plotted bar ± SEM.

Following challenge, the rBPI3Vc_mut_F2-HN2 vaccine treatment group shed the lowest amount of virus in nasal swabs compared to the virus detected in the Bovilis Nasalgen PI3 vaccinees as well as the sham treatment controls throughout the challenge period, although this was not statistically significant (**Figure 11A**). Notably, on day 3 post-challenge, shedding of the challenge virus was not detected in 1 out of 5 of the rBPI3Vc_mut_F2-HN2 vaccinees (**Figure 11A**). On day 6 post-challenge, peak mean shedding of the challenge virus by the rBPI3Vc_mut_F2-HN2 vaccinees (detected at 1:20 dilution) was lower than the peak titers detected in the Bovilis Nasalgen PI3 vaccinees (detected at 1:45 dilution) (**Figure 11A**). Viral shedding subsequently decreased in both groups and resolved by day 15. In contrast, the negative control group exhibited prolonged shedding of the challenge virus, with peak mean shedding occurring on day 9 post-challenge (**Figure 11A**). On day six post-challenge, the rBPI3Vc_mut_F2-HN2 vaccinees had lower viremia in blood than the Bovilis Nasalgen PI3 vaccinees and the sham negative controls (**Figure 11B**). Compared to the Bovilis Nasalgen PI3 vaccinees and the sham controls, the rBPI3Vc_mut_F2-HN2 vaccinees had lower, but insignificant, viremia in blood on day 6 (**Figure 11B**). On day 15 post-challenge, the rBPI3Vc_mut_F2-HN2 vaccinees had significantly (p=0.0223) lower viremia compared to the sham treatment group, but not the Bovilis Nasalgen PI3 vaccine treatment group (**Figure 11C**). Therefore, intranasal immunization of calves with the rBPI3Vc_mut_F2-HN2 prototype vaccine protected calves from severe disease and reduced viral loads in nasal secretions and peripheral blood.

### 3.8 Gross and histopathologic lesions

**Figure 12:**
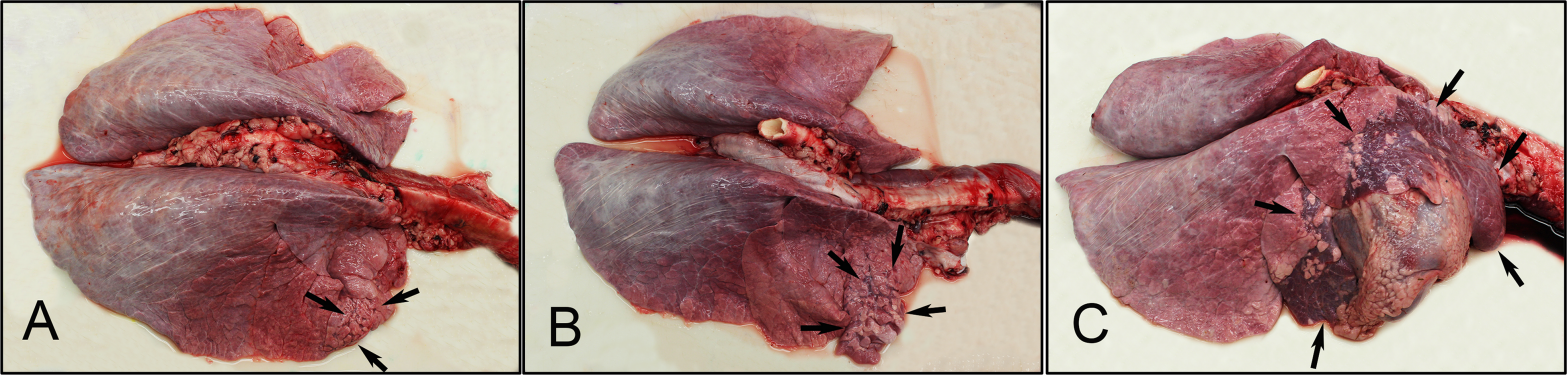
Gross lung lesions. Pulmonary atelectasis was the most common gross lesion observed consistently in all groups (black arrows), and the severity ranged from: **(A)** mild in the rBPI3Vc_mut_F2-HN2 vaccinees (10% of right cranial lung lobe affected); **(B)** moderate in the Bovilis Nasalgen treatment group (50% of right cranial lung lobe affected); and **(C)** severe in the sham (DMEM) control group (80% of right cranial and middle lung lobes affected).

The pulmonary lesions were overall mild but varied. Gross lesions identified in calves from this study included: none (grossly normal lung); patchy to coalescing atelectasis (3 calves in each of the rBPI3Vc_mut_F2-HN2 and the Bovilis Nasalgen PI3 vaccine treatment groups, 2 calves in the negative control group); mild fibrinous pleuritis or fibrous pleuritis (3 calves in each of the rBPI3Vc_mut_F2-HN2 vaccine treatment group and the negative control group, 2 calves in the Bovilis Nasalgen PI3 vaccine treatment group); mild pleural or pericardial effusion (30 ml fluid in # 5651, Bovilis Nasalgen PI3 vaccine treatment group); petechial hemorrhage of epicardium (1 calf in the Bovilis Nasalgen PI3 vaccine treatment group); and pleural hemorrhage (1 calf in the negative control group). The most common gross lesion was mild to severe cranioventral reddened and collapsed lung (atelectasis, 10% - 80% of parenchyma affected), which was present in all groups. Calf #5669 from the negative control group was the most severely affected animal (**Figure 12**).

**Figure 13:**
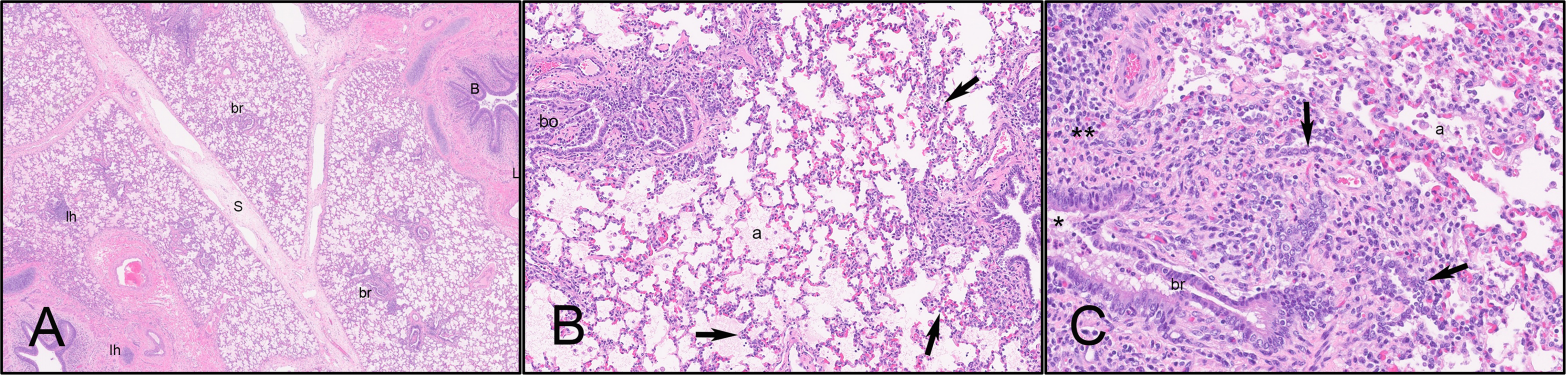
Histologic lesions. Histologic lesions observed in calf 5669 lung [sham negative control group] included **(A)** chronic interstitial pneumonia, expansion of interlobular septa (S), peribronchial inflammation (B), lymphoid hyperplasia (lh), alveolar edema, bronchiolitis obliterans (br), and expansion of interstitial septa. 2x magnification. **(B)** In more severely affected areas, there were poorly aerated alveolar spaces (a) containing edema and alveolar macrophages, bronchiolitis obliterans (bo), and chronic interstitial pneumonia (black arrows). 10x magnification. **(C)** In the most severely affected lobes, poorly aerated alveolar spaces (a) contained edema and alveolar macrophages, interstitial walls were expanded by inflammatory cells and occasionally are proliferative type 2 pneumocytes (black arrows); bronchioles (br) contained few neutrophils (*) and were surrounded by increased fibrous connective tissue and mixed inflammatory cells (**).

The most common histopathologic lesions observed in all groups included poor overall alveolar aeration (atelectasis), lymphoplasmacytic and histiocytic interstitial pneumonia, neutrophilic bronchitis and bronchiolitis, lymphoplasmacytic peribronchiolitis, bronchiolitis obliterans, and bronchial or bronchiolar associated lymphoid tissue hyperplasia. Importantly, the most severe histologic lesions were observed in calf 5669, one of the negative control calves, characterized by lymphoid hyperplasia, poorly aerated alveolar spaces with edema and alveolar macrophages, bronchiolitis obliterans characterized by partial to complete filling of bronchiolar lumens by fronds of proliferative fibrous connective tissue lined by airway epithelium, chronic interstitial pneumonia characterized by expansion of interstitial septa by lymphocytes and histiocytes, or proliferation of type 2 pneumocytes (**Figure 13**).

**Figure 14:**
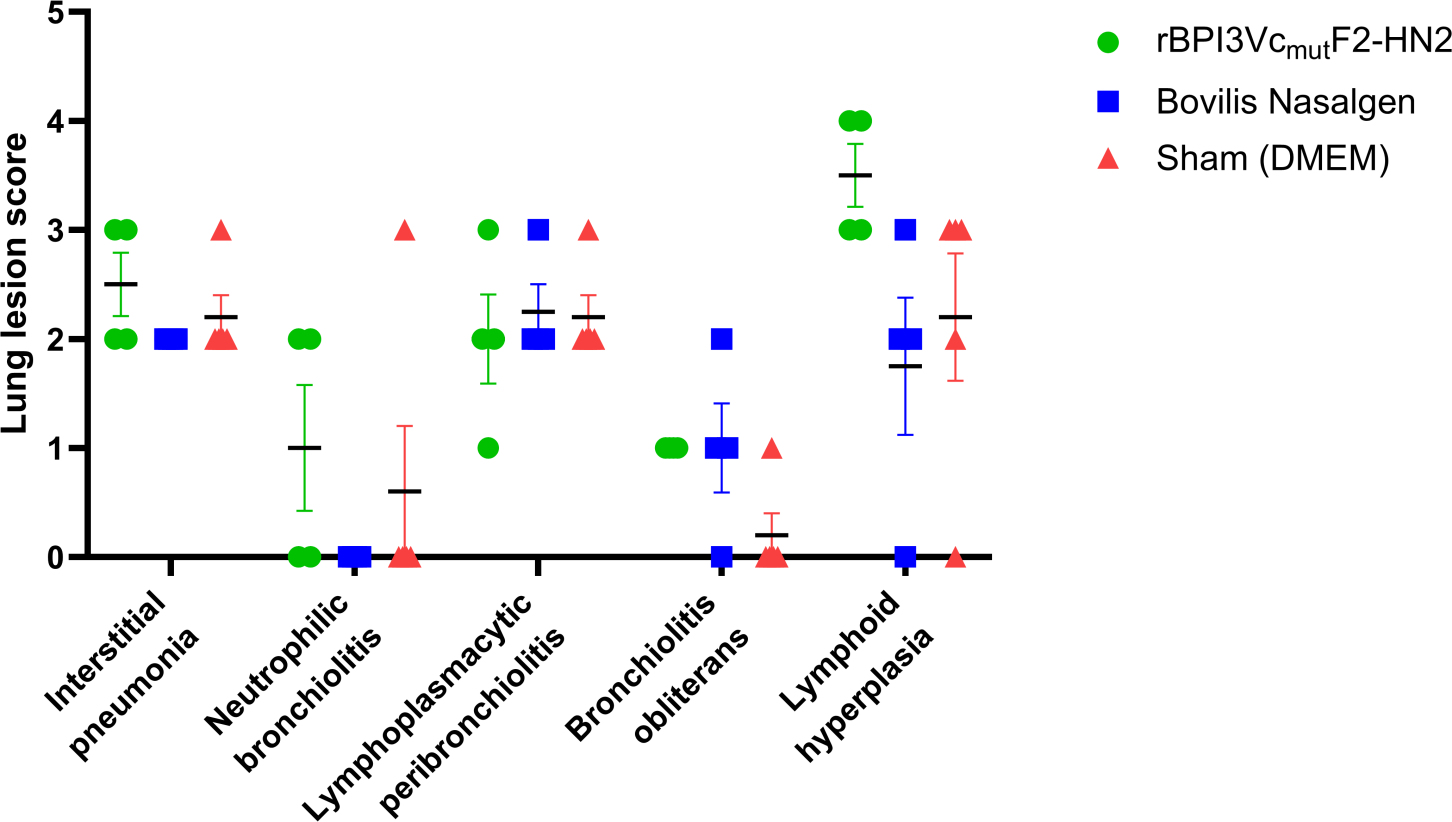
Histopathologic lung lesions. Lung lesions were scored from sections of the cranial, middle and caudal lobes of the right and left lung, based on the severity of lesions as follows; (0= normal; 1 = lesion is present; 2 = mild; 3 = moderate; 4 = severe). Individual lung lesion score for each animal is plotted. Bars represent mean lung lesion score for each group ± SEM.

Interestingly, tissue response and repair characterized by lymphoid hyperplasia and bronchiolitis obliterans, respectively (1), were more prominent in the rBPI3Vc_mut_F2-HN2 vaccine treatment group and the Bovilis Nasalgen PI3 vaccine treatment groups. However, no significant differences in gross and histologic lesions were observed between the three groups (**Figure 14**).

## 4 Discussion

Although live attenuated and killed BPI3V vaccines have been used for decades, concerns about their protective efficacy persist, partly due to antigenic variability. One key issue is that vaccine strains differ from circulating field strains, making commercially available vaccines effective against some, but not all, homologous and heterologous strains. In the United States, vaccine formulations have traditionally relied on live attenuated BPI3V genotype A strains, including SF-4 and Kansas/15626/84 strains (2, 5, 8–10). However, recent studies indicate that, in addition to genotype A, genotype B and C strains are also widely circulating in the U.S (5–7). Importantly, reference sera from SF-4 genotype A neutralized some, but not all, genotype A strains and showed lower titers against genotype B and C strains (5). Therefore, there is need for efficacious and broadly protective vaccines to improve the management of Bovine parainfluenza virus infections in cattle.

The primary objective of this study was to develop a BPI3V genotype C-based viral vector to create an attenuated recombinant prototype vaccine that safely protects cattle against diverse BPI3V genotypes A-C. The BPI3Vc TVMDL 16 reference strain was engineered to incorporate transgenes of interest, resulting in attenuated viral replication, a strategy known to reduce replication in non-segmented negative-strand viral vaccine vectors (41, 42). Insertion of the GFP gene downstream of the N gene generated a recombinant rBPI3Vc_mut_GFP virus with robust protein expression, confirming this site supports optimal transgene expression while preserving genetic stability (59). The rBPI3Vc_mut_GFP virus exhibited a temperature-sensitive attenuation phenotype similar to the established BPI3Va (Kansas/15626/84) vaccine strain, which is known for reduced replication in the lower respiratory tract of animals (60, 61). Live attenuated BPI3V vaccines developed since the 1970s are temperature-sensitive mutants (8, 51, 62). Consistent with the attenuation profiles of historical and current BPI3V vaccines, the rBPI3Vc_mut_GFP virus replicated efficiently at 37°C, moderately at 39°C, and was markedly inhibited at 41°C. In contrast, the wild-type BPI3Vc_wt_TVMDL16 virus replicated efficiently at all tested temperatures. These findings are consistent with previous studies demonstrating the suitability of the BPI3V backbone as a versatile vector for antigen delivery across multiple species (39, 59, 63–68). Temperature-sensitive attenuated viruses have been safely used in the U.S. and Europe as vaccine strains for mucosal immunization against PI3V and for developing live-vectored subunit vaccines (51, 59–61).

Although the BPI3V Fusion (F) and Hemagglutinin-Neuraminidase (HN) proteins are the main targets of neutralizing antibodies (22, 32, 40, 69), current commercial PI3 vaccines rely on naturally attenuated virus such as BPI3Va SF-4 and Kansas/15626/84 (7–9, 62). These vaccines typically offer protection against homologous genotype A strains but provide variable and suboptimal protection against heterologous BPI3V genotypes B and C (5, 7). Despite decades of use, these vaccines have not significantly reduced BPI3V prevalence in U.S. cattle herds, highlighting the need for more effective, cross-protective formulations (5–7). To address this gap, we engineered a multicistronic F2-HN2 antigen comprising novel F2 and HN2 polypeptides, derived from consensus sequences across BPI3V genotypes A-C, linked by a 2A autocleavable motif. This design aims to broaden epitope coverage and enhance cross-genotypic protection. The F2 sequence shared 74.1% identity with 62 BPI3V F protein sequences from genotypes A, B, and C, while the HN2 sequence showed 77.5% identity with 35 HN protein sequences from the same genotypes. Notably, F2 and HN2 retained high similarity to the F and HN proteins of vaccine strains BPI3Va SF-4 and Kansas/15626/84 (93.5% and 92.8% for F; 86.5% and 85.8% for HN), supporting compatibility with established immunogenic determinants while incorporating epitopes from diverse strains to enhance coverage. Consensus antigens designed to elicit broadly protective immunity have been successfully used to develop promising vaccine candidates (53, 55, 70–76). Compared to genotypes A and B, the F2 and HN2 sequences show greater identity, 95% and 100%, respectively, to genotype C F and HN proteins, reflecting the larger number of genotype C sequences available (29 of 62 F and 28 of 35 HN sequences). Additionally, when no clear consensus or dominant residue was present at a given position in the multiple sequence alignment, residues from the BPI3Vc_wt_TVMDL16 strain were preferentially selected, contributing to this bias. Importantly, the F2-HN2 multicistronic expression cassettes can be readily updated to incorporate protective determinants from emerging strains, offering a flexible platform for tailoring vaccine antigens to the most prevalent circulating variants.

The antigenic relevance of the expressed F2-HN2 proteins was confirmed by their recognition with anti-BPI3V polyclonal antibodies. Western blot analysis validated the functionality of the 2A autocleavable motif, demonstrating successful cleavage and expression of independent F2 and HN2 ectodomains on the surface of rBPI3Vc_mut_F2-HN2-infected cells. This separation supports optimal B cell recognition of both linear and conformational epitopes (77). Insertion of transgenes encoding either GFP (~0.8 kb) or F2-HN2 (~3.6 kb) between the N and P genes in the BPI3Vc_mut_ backbone had minimal impact on viral replication. This is consistent with prior findings demonstrating the capacity of the BPI3V backbone to generate stable recombinant viruses expressing heterologous transgenes (59, 64, 66, 77–80). The slightly reduced titers observed for rBPI3Vc_mut_GFP and rBPI3Vc_mut_F2-HN2 during the first three days post-infection suggest a modestly attenuated replication phenotype, consistent with attenuation profiles reported for other RNA virus-vectored vaccines (41, 60). Additionally, both the BPI3Vc_mut_ backbone and the F2-HN2 transgene remained stable over nine serial *in vitro* passages, with only two point mutations detected in the HN gene (K254R) and L gene (S2220Y, S2221L). This stability underscores the vector’s ability to maintain transgene integrity and expression over time, an essential feature of an effective viral vector (41, 81). Although *in vivo* studies are needed to assess the risk of reversion to virulence, no such events have been reported for existing commercial BPI3Va vaccines. Further research is warranted to identify the passage level at which mutations first arise and to determine their impact on viral replication.

The rBPI3Vc_mut_F2-HN2 prototype vaccine was well tolerated in five-month-old calves following intranasal administration, with no adverse effects on body temperature or weight gain compared to the commercial Bovilis Nasalgen^®^ IP (Merck) vaccine. It effectively primed both serum and mucosal IgG responses that strongly recognized representative wild-type BPI3V genotypes A-C. Notably, IgG levels induced by the prototype vaccine were significantly higher than those elicited by the commercial vaccine after the first booster, but not after the second, suggesting that a single booster may be sufficient to generate robust humoral immunity. Importantly, the rBPI3Vc_mut_F2-HN2 prototype vaccine elicited broadly neutralizing antibodies with exceptionally high virus neutralization titers (VNTs) against representative wild-type BPI3V genotypes A-C, significantly surpassing those induced by the commercial Bovilis Nasalgen^®^ vaccine (p<0.0001). This finding highlights the prototype vaccine’s potential to confer superior protection, addressing a critical gap in the current data regarding the breadth and potency of existing BPI3V vaccines against emerging heterologous strains in the U.S (2, 7, 11). In addition, existing reference sera exhibit low neutralizing titers against heterologous BPI3V strains (5). For example, the APHIS 475 BDV 0601 reference serum, derived from the BPI3Va SF-4 strain, demonstrates titers below 1:32 against BPI3V genotypes A-C (5), whereas sera from field infection studies have reported virus neutralization titers (VNTs) as high as 1:512 (82). In comparison, the VNTs elicited by the rBPI3Vc_mut_F2-HN2 prototype vaccine were markedly higher than those induced by experimental baculovirus-vectored HN (VNT: 1:96), adenovirus-vectored F (VNT: 1:256), and adenovirus-vectored FHN (VNT: 1:426) vaccines, demonstrating that the prototype vaccine induces a more potent and broader functional antibody response (22, 37). Interestingly, the strong BPI3Va-c-specific VNTs induced by the rBPI3Vc_mut_F2-HN2 prototype vaccine were primarily driven by the F2-HN2 antigens, as evidenced by significantly lower VNTs in animals immunized with the vector control construct (rBPI3Vc_mut_TMSP), which were comparable to those elicited by the commercial vaccine. This suggests that while the vector backbone contributes modestly to VNTs, the enhanced response is largely attributable to the F2-HN2 insert, potentially due to its proximal position to the promoter and/or codon optimization enhancing antigen expression. Additionally, the novel F2 and HN2 glycoproteins incorporated into the prototype vaccine are likely functional in viral attachment and entry, potentially exposing a broader array of conserved conformational epitopes across BPI3V genotypes A-C than those found in the genotype C-derived F and HN proteins. Sequence analysis of F2 and HN2 supports their immunogenic potential: F2 substitutions are primarily clustered at the N-terminus (within the cleaved signal peptide) and the C-terminal cytoplasmic domain, whereas HN2 substitutions are more widely distributed across the protein. This pattern suggests that HN2 may play a greater role in enhancing neutralization breadth, while F2 and HN2 together contribute synergistically to eliciting potent, broadly neutralizing antibodies.

Intranasal immunization of calves at an early age can effectively prime immune responses, enabling the onset of protective immunity as early as one week of age, even in the presence of maternally derived antibodies (1, 24, 27). Immunization of calves with the rBPI3Vc_mut_F2-HN2 prototype vaccine elicited strong mucosal and systemic antibody responses, which are desirable for preventing infection at mucosal entry sites and mitigating disease severity in the event of breakthrough infection (1, 12, 13). BPI3V-specific neutralizing titers of 1:32 are generally considered protective (1, 11–13). Although the duration of immunity from a single dose of the bivalent Bovilis Nasalgen^®^ IP used in this study is not established, the related pentavalent Bovilis Nasalgen^®^ 3-PMH provides protection for only up to three months (83). Therefore, the optimal dosing regimen and duration of immunity conferred by the rBPI3Vc_mut_F2-HN2 prototype vaccine must be empirically determined.

Challenge induced only mild clinical signs, with most calves maintaining rectal temperatures below 103.1°F (normal range: 101–103°F), and temperatures exceeding 103.5°F, indicative of inflammation (84), were infrequent. Consistent with prior studies (25, 26), no significant differences in pyrexia were observed between vaccinated and non-vaccinated calves. Although all calves gained weight during the challenge phase, the negative control group showed a markedly lower rate of weight gain compared to vaccinated groups. Unexpectedly, all groups exhibited decreases in leukocyte and platelet counts; however, calves immunized with the rBPI3Vc_mut_F2-HN2 vaccine demonstrated a more rapid recovery. Importantly, the observed declines remained within normal reference ranges for healthy calves: 4,700-11,400/µl for WBCs and 200–590 K/mm³ for platelets (58). Sporadic platelet clumping was observed, which may have influenced platelet counts. There is limited data linking BPI3V infection in cattle to leukopenia or thrombocytopenia, underscoring the need for future studies comparing the leukocyte profiles of challenged and non-challenged calves under similar husbandry conditions. Notably, calves immunized with the rBPI3Vc_mut_F2-HN2 prototype vaccine exhibited the lowest nasal virus shedding and reduced viremia throughout the challenge phase. In contrast, negative control calves exhibited prolonged virus shedding, with peak levels on day 9, and higher viral loads in blood. These findings demonstrate that intranasal immunization with the rBPI3Vc_mut_F2-HN2 prototype vaccine effectively reduced viral replication in both blood and nasal secretions, providing protection from severe disease, consistent with outcomes reported for other intranasally delivered BPI3V vaccines (13, 24, 26, 27).

Gross and histopathologic lung lesions in challenged calves were consistent with acute BPI3V infection (1, 56). Mild lesions, primarily atelectasis, were observed in calves vaccinated with rBPI3Vc_mut_F2-HN2, whereas moderate and severe lesions were noted in the Bovilis Nasalgen and negative control groups, respectively, aligning with findings from previous studies (26).

Histopathologic analysis confirmed hallmark BPI3V lesions, interstitial pneumonia, neutrophilic bronchiolitis, and lymphoplasmacytic peribronchiolitis, across all treatment groups. While differences in lesion severity were not statistically significant, the negative control group exhibited slightly higher disease scores, particularly calf 5669, which showed severe interstitial pneumonia, underscoring the clinical impact in non-vaccinated cattle. Elevated lymphoid hyperplasia in rBPI3Vc_mut_F2-HN2 vaccinees and bronchiolitis obliterans in both vaccinated groups may reflect reparative processes in the airway epithelium occurring around 14 days post-infection, characterized by epithelial hyperplasia and, in some cases, fibroplasia and neovascularization of the bronchiolar connective tissue (bronchiolitis obliterans) following injury (1, 56).

The data presented here establish a foundation for advancing the management of bovine parainfluenza virus infections. Unlike traditional MLVs, subunit vaccines offer the flexibility to incorporate antigens from evolving strains. Recent studies have shown that replication-incompetent adenovirus-vectored vaccines expressing BPI3V F or combined F and HN proteins can elicit neutralizing antibodies, stimulate splenic CD3^+^/CD8^+^ T cell proliferation, and induce both pro- and anti-inflammatory cytokines (IFN-γ and IL-4) (22, 37). Similarly, intranasal delivery of nanoparticle-encapsulated whole-virus BPI3V antigens elicited stronger mucosal antibody responses than a commercial intranasal vaccine (85), while a baculovirus-vectored HN vaccine induced neutralizing antibodies that conferred protection upon challenge (32). Notably, virus neutralization (VN) titers in these studies were generally below 1:426. In contrast, the current study demonstrates substantially higher and broadly protective VN titers against BPI3V genotypes A-C (1:4096 - 1:8192), surpassing those reported for earlier subunit vaccine candidates and commercial MLVs in North American and European studies (11, 24, 25, 50). Recent European efforts have optimized MLV delivery, showing that intranasal vaccination of young calves can induce mucosal immunity and provide early protection, even immediately after birth (26) or by one week of age (27), despite the presence of maternal antibodies (24). However, such safe and effective intranasal vaccines remain untested in neonatal calves in North America, positioning the rBPI3Vc_mut_F2-HN2 prototype vaccine as a strong candidate for further evaluation in this population.

In conclusion, this study demonstrates the successful development of a BPI3Vc vector with unique cloning sites, enabling the generation of recombinant live-vectored prototype vaccines. The platform offers flexibility for future refinement to enhance broad protection against diverse BPI3V strains. Using this platform, a multicistronic expression cassette encoding novel F2-HN2 polypeptides, derived from consensus sequences of BPI3V genotypes A-C, was generated. The resulting rBPI3Vc_mut_F2-HN2 prototype vaccine effectively displayed these antigens on the surface of infected cells, facilitating optimal B cell recognition *in vivo*. Importantly, intranasal immunization of calves with the rBPI3Vc_mut_F2-HN2 prototype vaccine induced potent, broadly neutralizing antibodies that reduced wild-type virus shedding following challenge. The robust neutralizing response was driven by the F2-HN2 antigens. This versatile vaccine platform offers opportunities to develop customized, contemporary vaccines not only for BPI3V but also for other BRD-associated pathogens such as BVDV.

## Data Availability

The data presented in the study are deposited in the Dryad repository: https://doi.org/10.5061/dryad.gtht76j27.
